# Climatic imprint on interfacially controlled platinum–palladium resources

**DOI:** 10.1093/pnasnexus/pgag196

**Published:** 2026-06-01

**Authors:** Emily G Wright, Ivey Wang, Yihang Fang, Elaine D Flynn, Jeffrey G Catalano

**Affiliations:** Department of Earth, Environmental, and Planetary Sciences, Washington University in St.Louis, Saint Louis, MO 63130, USA; Department of Earth, Environmental, and Planetary Sciences, Washington University in St.Louis, Saint Louis, MO 63130, USA; Department of Earth, Environmental, and Planetary Sciences, Washington University in St.Louis, Saint Louis, MO 63130, USA; Department of Earth, Environmental, and Planetary Sciences, Washington University in St.Louis, Saint Louis, MO 63130, USA; Department of Earth, Environmental, and Planetary Sciences, Washington University in St.Louis, Saint Louis, MO 63130, USA

**Keywords:** critical minerals, platinum group elements, weathering, climate, interfacial processes

## Abstract

Iron oxide–rich laterites, soils, and regolith formed from the weathering of ultramafic rocks represent untapped unconventional resources for the critical minerals platinum (Pt) and palladium (Pd), but the fundamental surficial geochemistry of these elements remains poorly understood. Depletion of Pd relative to Pt occurs in some weathering zones in semi-arid climates. The accepted model attributes this Pt–Pd chemical fractionation to preferential complexation of Pd by dissolved chloride (Cl). However, similar fractionation is not observed in laterites of humid equatorial regions despite substantial wet deposition of Cl. The established mechanistic model for Pt and Pd behavior during weathering thus inaccurately predicts the distribution of these critical minerals in many settings, hindering global resource assessment. We show through mineral-fluid partitioning experiments coupled to element-specific spectroscopy that this canonical explanation for Pt–Pd fractionation is invalid: Cl complexation does not differentially mobilize Pd versus Pt. Instead, mineral-specific interfacial reactions control Pd and Pt accumulation. Modeling of Pt–Pd fractionation in representative weathering zone profiles demonstrates subequal retention in goethite-rich settings and Pd depletion in hematite-rich zones, accurately predicting trends observed in soils and laterites. Iron oxide mineralogy, reflecting modern and past regional climate conditions, is likely the primary determinant of Pt and Pd endowment in weathering zone resources. This new model for Pt and Pd mobilization and accumulation behavior provides a mechanistic foundation for exploration and recovery of Pt group elements from ultramafic regolith deposits.

Significance statement Platinum (Pt) and palladium (Pd) accumulation in regolith is a potential future source of these critical minerals, but an incomplete understanding of their fundamental geochemistry prevents accurate resource prediction. Dissolved chloride (Cl) is hypothesized to preferentially leach Pd during weathering. However, despite rain being a primary input of Cl, this leaching often does not occur in humid equatorial settings. We show that Cl cannot preferentially mobilize Pd and instead demonstrate that mineral-specific interfacial reactions control the behavior of these elements. (Paleo)climate-controlled regolith mineralogy drives global spatial patterns of Pt–Pd separation and the extent of their accumulation in soils and laterites. Our climate–mineralogy model for Pt and Pd distribution in regolith provides foundational insight needed to predict and identify new critical mineral resources.

## Introduction

The critical minerals platinum (Pt) and palladium (Pd) are essential for clean energy technologies, electronics, and defense applications but have vulnerable supply chains (1, 2). Weathering zones formed from ultramafic rocks, including major Pt group element (PGE) ore deposits, contain elevated concentrations of Pt and Pd, ranging from tens of μg/kg to as high as 5 mg/kg (3–6). These soils, laterites, and oxidized ores represent promising untapped unconventional resources that often contain Pt and Pd concentrations similar to primary ores under active production (1, 7). Laterite formation often generates bulk PGE concentrations greater than the underlying parent rock (5, 8). Mobilization and accumulation processes in weathering zones also underlie geochemical prospecting for primary PGE deposits (4, 9), generate placer deposits (6, 10), and potentially enable Pt and Pd phytomining (11, 12). However, the mechanisms controlling Pt and Pd mobility and accumulation during weathering are poorly characterized, hindering prediction of the occurrence and resource potential of regolith-hosted PGE deposits.

Weathering zones formed from ultramafic PGE occurrences in some semi-arid regions display depletion of Pd relative to Pt (4, 6). This differential mobility has been classically explained by enhanced chloride (Cl) complexation of Pd compared with Pt, driving preferential Pd leaching (4). However, the preferential loss of Pd is not observed in humid-region laterites in Indonesia and the Dominican Republic (5, 13) despite the elevated wet deposition of Cl because of proximity to the ocean (14). These regional patterns of Pt–Pd fractionation are inconsistent with the accepted mechanism, suggesting this model for PGE mobility is invalid and impeding predictions of resource potential. Additionally, substantial disagreement regarding the stability of Pd and Pt complexes (15, 16) generates over four orders of magnitude variations in predicted aqueous speciation (Fig. S1). This large uncertainty hinders prediction of Pt and Pd solubilization during weathering, further undercutting the applicability of the established mechanistic model.

Reactive secondary minerals inhibit leaching during weathering via surface complexation reactions (17). Interfacial reactions with iron oxides likely play an important role in Pt and Pd behavior during weathering because these phases are highly abundant in laterites (up to 92 wt.%) (18) and have high specific surface areas (19). Goethite and hematite are the most common iron oxides in such systems (18, 20, 21) and their relative abundances are controlled by climate (22–24). This climate–mineralogy relationship is foundational to established paleoclimate approaches (25, 26). However, the interfacial geochemistry of Pt and Pd associated with iron oxides is largely unexplored. We recently demonstrated that Cl enhances both Pd(II) mobilization through aqueous complexation and retention via Pd-Cl ternary surface complexation at iron oxide-solution interfaces for hematite (α-Fe_2_O_3_) and ferrihydrite (∼Fe_10_O_14_(OH)_2_) (27). Interfacial reactions partially offset the solubilizing effects of Cl complexation, and accurately predicting Pd mobility requires accounting for both processes. Similar effects of solution and interfacial processes on Pt mobility have not been investigated for conditions relevant to weathering zones. Additionally, the interfacial reactivity of Pt and Pd with goethite (α-FeOOH), abundant in weathering zones (20, 28), is unknown. While iron oxides are known to accumulate substantial quantities of Pt and Pd in ultramafic weathering zones (3), the general lack of correlated mineralogical information prevents identification of the controlling absorbent phase(s). Determining the impact of Cl on Pt and Pd interfacial reactions with these major weathering products is necessary for creating a predictive model of the resource occurrence of these critical minerals. This enhanced knowledge of chemical controls on PGE mobility would also have ancillary benefits, from improving prediction of Pt and Pd fate in diverse environmental settings to serving as a foundation for developing in situ leaching methods.

Here, we present the first comprehensive assessment of Pd(II) and Pt(II) adsorption to hematite and goethite under weakly acidic conditions in order to test whether Cl complexation can cause differential mobility of these critical minerals during weathering. We coupled macroscale solid–fluid partitioning experiments under systematically varying dissolved Cl concentrations with element-specific X-ray absorption spectroscopy measurements of Pt and Pd interfacial coordination. This experimental approach allowed us to isolate the effect of Cl and directly measure surface speciation, which is not possible (29) with the low PGE concentrations of natural samples (3–6). Through these studies, we demonstrate that mineral-specific interfacial reactions control Pt and Pd mobility. We then develop a model for Pt–Pd fractionation in weathering zones controlled by iron oxide mineralogy, a critical environmental parameter reflecting local (paleo)climate conditions (22–24). This model reproduces distinct depth trends in Pt/Pd ratios observed in soils, laterites, and regolith formed from ultramafic rocks (13, 30–32).

### Mineral- and Cl-specific impacts on Pt and Pd retention

**Figure 1:**
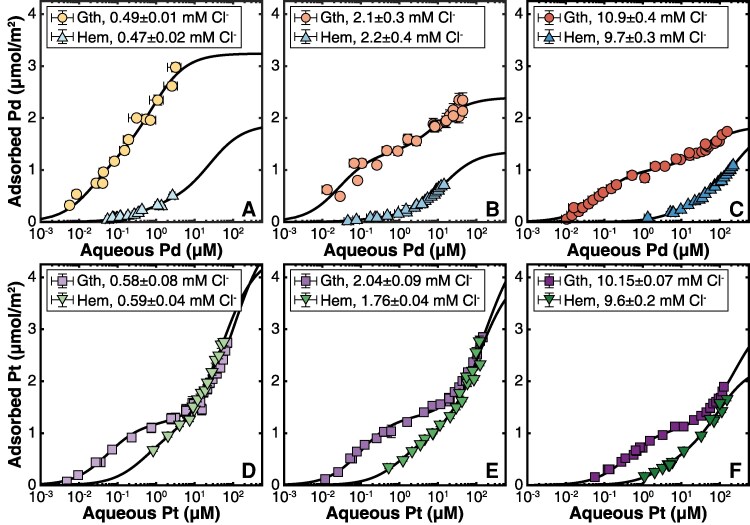
Impact of fluid composition on Pd and Pt retention by iron oxides. The amount of (A–C) Pd and (D–F) Pt adsorbed to goethite and hematite (per square meter of mineral surface area) compared with the final dissolved Pd or Pt at pH 4 ± 0.1 in the presence of varying amounts of dissolved Cl. The effects of mineralogy are compared for adsorption in approximately (A, D) 0.5, (B, E) 2, and (C, F) 10 mM total dissolved Cl. For many data points, experimental uncertainties are smaller than the symbol size and not visible. Corresponding Langmuir isotherm fits to the partitioning data (Table S2) are shown as solid lines. A subset of each Pt-goethite dataset has been corrected for systematic differences in surface area between multiple experiments; see the Supplementary Information, Fig. S22, and Table S2 for details. The data for Pd adsorption to hematite were previously published (27) but were refit with two-component Langmuir isotherms (Table S2).

We performed mineral–water partitioning experiments to quantify Pt(II) adsorption to both hematite and goethite and Pd(II) adsorption to goethite at pH 4 ± 0.1 in fluids with sequentially increasing Cl concentrations, complementing our previously reported data for Pd(II) adsorption to hematite (27). The mineral sorbents were synthesized and characterized using standard procedures (Fig. S2; Table S1) (19). Binding affinities of Pt and Pd are greater for goethite than for hematite (Fig. 1). For Pt, partitioning behaviors for both minerals converge at elevated dissolved Pt concentrations, yielding similar maximum binding capacities (Fig. 1). Hematite consistently adsorbs less Pd than Pt, with both lower affinity and binding capacity (Figs. 1 and S3). However, this relationship inverts for goethite, with Pd displaying strong adsorption affinity distinct from the PGE–mineral pairs studied (Fig. 1). These observations demonstrate substantial mineral-specific differences in solid–water partitioning behavior for Pd compared with muted variations for Pt.

Increasing Cl concentrations systematically inhibit adsorption for both critical elements (Fig. 1). Binding suppression by Cl is of a similar magnitude for Pt and Pd on hematite (Fig. S4; Table S2) and does not result from changes in ionic strength (Fig. S5) or surface charge (Table S3). Increasing aqueous complexation by Cl thus similarly solubilizes both Pt and Pd from hematite, inconsistent with all models of Pt(II) speciation in Cl-bearing fluids (Fig. S1), which predict weak complexation. The effects of Cl on Pt and Pd binding to goethite are muted at low Cl concentrations, with Pd showing the greatest retention (Fig. S4). While relative Pd mobilization from goethite is greater than for Pt in response to increasing Cl concentrations at high surface coverages, these conditions involve dissolved PGE concentrations exceeding levels in natural systems (9). At lower, environmentally representative dissolved Pt and Pd concentrations, both elements show similar mobilization from goethite by Cl (Fig. S4). These findings demonstrate that existing thermodynamic data for dissolved Pt(II) species are incomplete and likely flawed, hindering prediction of potential Pt resources created by weathering. Further, observed adsorption behaviors contradict the canonical interpretation of differential Pd and Pt mobility during weathering resulting from the distinct strengths of Cl complexation. Instead, while there may be differences in the aqueous speciation of PGEs (Fig. S1), it is primarily mineral-specific interfacial reactions that control the mobility and accumulation of Pd and Pt.

### Interfacial coordination drives mineral-specific Pt(II) adsorption patterns

**Figure 2:**
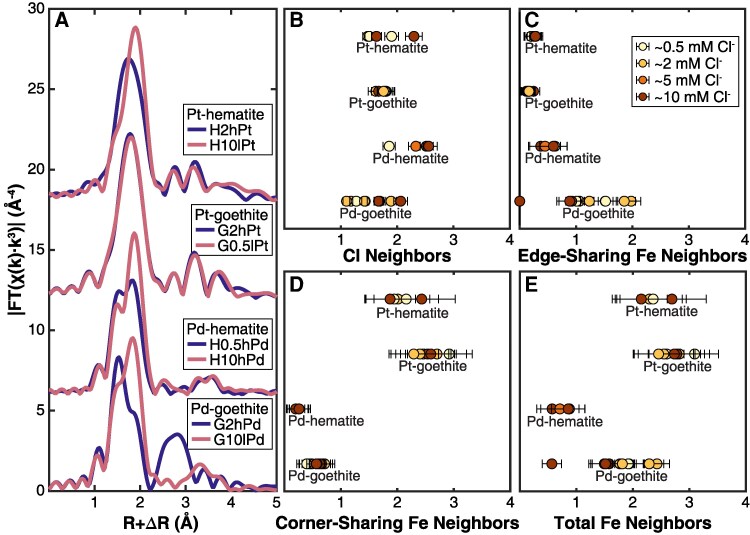
Variability in the interfacial coordination of Pd and Pt. A) Representative magnitudes of Fourier transform EXAFS spectra of Pd(II) and Pt(II) adsorbed to goethite and hematite under a range of dissolved Cl concentrations. See Table S4 for specific sample conditions. Data were obtained at the Pd K-edge and Pt L_3_-edge. B–E) Comparison of major features in the EXAFS-derived interfacial coordination of Pd and Pt: B) number of Cl ligands coordinated to Pd and Pt, with the balance of these four-coordinated species bound to oxygen; C) number of neighboring Fe atoms at <3.2 Å resulting from Pd or Pt coordinating to the edges of iron octahedra on the goethite or hematite surfaces; D) number of neighboring Fe atoms at >3.6 Å resulting from Pd or Pt coordinating to the corners of iron octahedra; and E) total number of neighboring Fe atoms. Full fitting results are reported in Tables S7, S8, and S10. Spectra and fitting-derived coordination information for Pd adsorbed to hematite were previously published (27) and are additionally reported in Table S11.

Mineral-specific adsorption affinities (Fig. 1) and responses to Cl addition (Fig. S4) suggest that Pt(II) displays distinct binding mechanisms to goethite and hematite. We applied extended X-ray absorption fine structure (EXAFS) spectroscopy to assess Pt(II) coordination on the surfaces of these minerals (Figs. 2 and S6; Table S4). Principal component analysis (PCA) indicates that Pt(II) surface speciation on goethite is invariant under the Pt(II) and Cl concentrations investigated (Fig. S7; Table S5). In contrast, two components are present in the spectral series for Pt(II) bound to hematite (Fig. S8; Table S6). This variance reflects changes in the number of Cl ligands coordinating Pt on the hematite surface with increasing dissolved Cl concentration and Pt(II) surface coverage (Table S7). For goethite, coordination to Cl was statistically invariant among the samples studied (Table S8). On both minerals, neighboring Fe atoms occur at 3.0–3.1, 3.7, and 3.9 Å for adsorbed Pt(II) species (Tables S7 and S8). These distances correspond to Pt(II) binding to the edges (3.0–3.1 Å) or corners (3.7 and 3.9 Å) of iron octahedra on the mineral surfaces. A large number of potential Pt(II) surface configurations are possible (Figs. S9 and S10) that cannot be further distinguished because of ambiguity from radial-averaging intrinsic to EXAFS spectra. The invariance in the number of neighboring Fe atoms (Figs. 2 and S11) indicates that Pt(II) binding geometries are indistinguishable on the goethite and hematite surfaces. The interfacial chemistry generating distinct Pt(II) macroscopic adsorption to goethite and hematite thus primarily reflects different stabilities of Cl ternary surface complexes. The ubiquitous occurrence of Pt-Cl surface species cannot be reconciled with the predicted weak complexation of Pt(II) by Cl in solution (Fig. S1), further questioning the validity of available thermodynamic data.

### Origin of Pd(II) hyperaccumulation by goethite

While Pt(II) and Pd(II) show enhanced binding to goethite compared with hematite (Fig. 1), the magnitude of their mineral-specific absorption affinities diverges. Goethite hyperaccumulates Pd(II), with 10 to 100 times greater adsorption affinities and double the maximum binding capacity of hematite (Table S2). Similarly, Pd(II) adsorption affinities for goethite are two to five times greater than displayed by Pt(II). At the low dissolved concentrations expected in weathering environments (9), goethite, if present, will predominantly control both absolute and relative Pt and Pd mobility and accumulation, regardless of Cl concentration.

We again applied EXAFS spectroscopy to identify the molecular origins of strong Pd(II) adsorption to goethite compared with weaker binding to hematite (Fig. 2). A PCA of all spectra of Pd(II) bound to goethite indicates the presence of two components (Fig. S12; Table S9). The EXAFS spectra vary systematically within two regions of the Fourier transform: 1.0–2.1 and 2.3–3.2 Å (Figs. 2, S13, and S14). This reflects changes in coordination to Cl ligands and the number of Fe neighbors at the mineral surface (Table S10). Both of these parameters vary systematically with Pd(II) surface coverage but are only weakly affected by dissolved Cl concentration (Table S10). These spectral variations demonstrate that two distinct Pd(II) species form on the goethite surface, corresponding to the bimodal adsorption affinities observed under all conditions (Fig. 1; Table S2).

At lower surface coverages on goethite, Pd is consistently coordinated to ∼2 Cl ligands irrespective of dissolved Cl concentration (Table S10). Unlike for Pd adsorption to hematite (Table S11) (27), there is no clear trend between the number of coordinating Cl atoms and the dissolved Cl concentration (Fig. 2). The resulting Pd-Cl ternary surface complexes bind primarily to corners of iron octahedra on the goethite surface, as shown by the correlation between the number of Cl ligands and neighboring Fe atoms at 3.6 Å (Fig. S15). Multiple possible surface configurations are consistent with such Pd coordination (Fig. S9) which cannot be unambiguously distinguished by EXAFS spectroscopy. This ternary Pd-Cl species corner-sharing to surface octahedra creates the anomalously high Pd(II) binding affinity to goethite.

Increasing Pd(II) surface coverage on goethite results in systematic replacement of Cl ligands by O neighbors (Table S10) originating from H_2_O ligands, OH ligands, or surface O sites. In addition, a larger Fourier transform feature between 2.3 and 3.2 Å (Figs. S13 and S14) associated with an increased number of Fe neighbors at short distances (2.9 and 3.2 Å) indicates substantial Pd(II) binding to the edges of iron octahedra. These trends in the abundances of adjacent Cl, O, and Fe atoms are strongly correlated (Fig. S15), demonstrating that Pd(II) species highly coordinated to the goethite surface at elevated surface coverage have minimal binding to Cl, even at high dissolved Cl concentrations. The number of and distances to Fe neighbors for this highly coordinated species are consistent with Pd(II) substituting into chains of Fe octahedra (Fig. S16). Further, the EXAFS spectra are inconsistent with the formation of Pd hydroxy polymers (Fig. S17) and transmission electron microscopy (TEM) demonstrates the absence of a Pd precipitate (Fig. S18). Such surface incorporation of Pd(II) stands in strong contrast with its binding to hematite, where adsorbed species have low coordination to surface iron octahedra (Fig. 2) and speciation does not substantially shift as a function of surface coverage (Table S11) (27). The substantially greater Pd(II) adsorption capacity of goethite compared with hematite, and the second surface species formed, thus results from Pd uniquely incorporating into the goethite structure. The dense hematite structure contains a nonlinear, 3D arrangement of adjacent Fe octahedra, a configuration likely unfavorable for incorporating square-planar Pd(II). In contrast, Pt(II) does not incorporate into the goethite surface despite its similar size and coordination chemistry (33). Inhibition from entering the goethite surface structure may result from Pt(II) forming distinct, stable adsorbed species or a stronger affinity for retaining Cl ligands.

Iron vacancy surface defects, common for goethite, are known to impact interfacial reactivity (34–36) and have been previously hypothesized to cause irreversible trace metal binding after aging (37). We assessed the role of these defects in promoting the high binding affinity of Pd(II) through additional experiments using hydrothermally annealed goethite (Fig. S19), a treatment that removed these vacancies (36). However, annealing had no impact on Pd(II) adsorption (Fig. S20) and surface speciation did not change (Fig. S12); Pt(II) binding was similarly unaffected (Figs. S6, S11, and S20). The observed hyperaccumulation of Pd(II) by goethite is thus driven by a high-affinity Pd-Cl surface species and the presence of distinct, highly coordinated complexes formed via entrapment in chains of Fe octahedra, not by binding at Fe vacancy surface defects. Surface incorporation of Pd(II) may instead be promoted by labile Fe(III) surface sites, which should readily form under our experimental conditions and are more easily perturbed compared with the bulk structure (38). Pd(II) bound via surface incorporation may resist remobilization, inhibiting recovery via phytomining (11, 12) without pretreatment to dissolved goethite to enable plant uptake.

### Iron oxide mineralogy drives differential mobility of Pt and Pd in the environment

**Figure 3:**
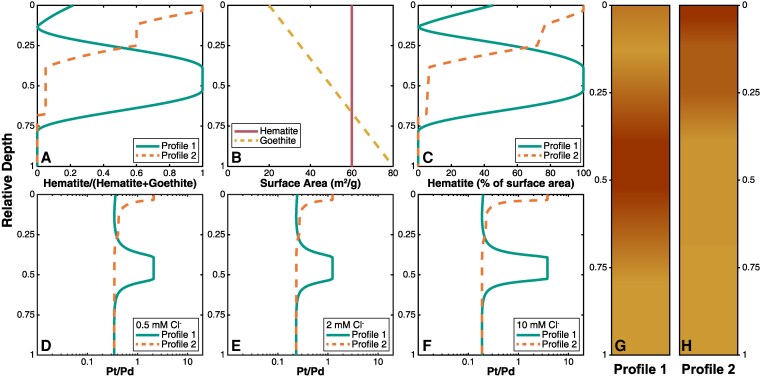
Model of Pt–Pd fractionation in climatically distinct weathering zones. A) Iron oxide mineralogical trends in the two profiles modeled. B) Surface areas of hematite and goethite as a function of relative depth (used for modeling both profiles). C) Relative abundance of hematite with depth in both modeled profiles as a function of total surface area. D–F) Calculated Pt/Pd in approximately (D) 0.5, (E) 2, and (F) 10 mM total Cl. G) Profile 1 represents a scenario of yellowing toward the surface and thus a more humid climate. H) Profile 2 represents a scenario of reddening and lower available moisture.

Our new findings demonstrate that both iron oxide mineralogy and dissolved Cl impact Pt and Pd retention in weathering zones (Fig. 1). To better evaluate how these factors influence absolute PGE retention, as well as Pt–Pd fractionation, we developed weathering profile models (Fig. 3) using parameters derived from our mineral–water partitioning results (Table S2). Goethite and hematite have distinct binding affinities for both Pt and Pd driven by variations in surface speciation. We considered two representative laterite profiles based on literature reports of quantitative mineralogy versus depth (e.g. 18, 39), supplemented by qualitative observations of mineralogy in additional laterites (40–42). In yellowing laterites, goethite content increases toward the surface, with hematite locally abundant at depth (39, 40, 42); in reddening laterites, hematite formation is favored near the surface, with goethite present deeper in the profile (18, 21). The typically high abundance of hematite and goethite (up to 92 wt.% combined) (18, 21, 39) and their high specific surface areas (19) indicate that these phases should dominate interfacial reactivity and thus PGE behavior in laterites and weathered ore zones. Other mineral phases could contribute second-order modifications of predicted retention; further discussion is provided in the Supplementary Information. Direct simulation of specific laterites is not possible because there is not correlative data on quantitative mineralogy and PGE concentrations; this limitation is a fundamental constraint applicable to all potential modeling approaches.

Applying low dissolved Pd and Pt concentrations (∼2 ng/L) representative of water near a PGE ore deposit (9), the model predicts elevated iron oxide–associated concentrations (10^−1^ to 10^1^ mg/kg) of both PGEs (Fig. S21). For combined hematite and goethite abundances of 30–90 wt.%, typical of iron oxide–rich weathering profiles (18, 21, 39), the resulting solid-phase Pd and Pt concentrations in our simulations are similar to PGE abundances measured in laterites (20, 43). Additionally, these concentrations lie within the range of grades of known ore deposits (1).

Our model demonstrates that Cl has a weak effect on Pt–Pd fractionation (Fig. 3), contrasting with the canonical explanation for differential mobilization of these PGEs during weathering (4). The subtle, second-order impacts of variations in Cl concentrations (Fig. 3) cannot be responsible for observed depth-dependent trends in Pd depletion. This insensitivity to Cl may have a distinct origin for each PGE. Strong Cl complexation of Pd (Fig. S1) is offset by enhanced formation of Pd-Cl ternary surface complexes as Cl increases (Fig. 2). In contrast, Pt displays substantial but largely invariant ternary surface complexation (Fig. 2) that may not counteract solubilization from predicted weaker aqueous Cl complexation (Fig. S1). The lack of a valid aqueous speciation model for Pt, foundational knowledge long-established for most elements, hinders further assessment and precludes application of reactive transport models to simulate Pt–Pd fractionation under a wider range of conditions. Irrespective of these mechanistic uncertainties, differential Cl complexation cannot cause the Pt–Pd fractionation observed in weathering zones. Instead, our model demonstrates that iron oxide mineralogical variability, specifically the ratio of hematite to goethite, primarily creates substantial variations in PGE retention and fractionation (Fig. 3).

Across both profiles, our model simulates subequal Pt/Pd ratios in goethite-rich zones, while Pd is preferentially lost in hematite-dominated zones (Fig. 3). These predictions align with observations from both hematite- and goethite-rich laterites. The presence of hematite is generally correlated with an increase in the Pt/Pd ratio, both at depth (profile 1) and near the surface (profile 2) (13, 32). In contrast, transitions to a more goethite-rich zone result in a decrease in the Pt/Pd ratio (30, 31), consistent with model predictions (Fig. 3). While not directly simulated by our model, observations of greater Pd depletion in hematite-rich nodules compared with a hematite-poorer matrix within the same zone (32) are consistent with its demonstrated mineralogical control on fractionation. Beyond fractionation trends, the model predicts the preferential retention of both PGEs where goethite is most abundant (Fig. S21), consistent with Pd and Pt concentrations increasing (and sometimes peaking) in goethite-rich zones within laterites (5, 13, 30).

### (Paleo)climatic controls on iron oxide mineralogy influence Pt/Pd fractionation

The goethite/hematite ratio in laterites is a primary determinant of Pt and Pd retention, as well as their fractionation. The relative abundance of these two phases has long been demonstrated to reflect soil moisture status, controlled by climate (22, 23). Cooler and more humid environments favor goethite, while hematite is often the dominant mineral in drier, hotter environments (22, 44). The goethite/hematite ratio in soils is strongly correlated with modern precipitation (25) and has been used by the paleoclimate community to reconstruct past climatic conditions (25, 26). Since the formation of laterites involves intense chemical leaching over millions of years, the current composition and zonation of laterites may reflect previous climatic regimes (45). Plate tectonics has driven the movement of continents over this timespan, with paleoclimates producing distinct mineralogy compared with what would form under current conditions (24, 46).

**Figure 4:**
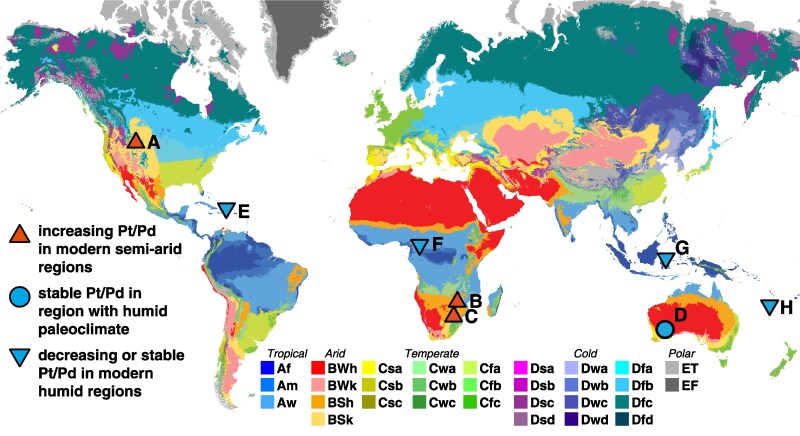
Climatic variation in Pt–Pd fractionation in ultramafic weathering zones. Location of laterites, classified by Pt/Pd ratio trends toward the surface, compared with global Köppen–Geiger climate classifications for 1991–2020 (47): A) soils above the Stillwater Complex in Montana, United States of America (4); B) oxidized ores of the Great Dyke in Zimbabwe (3); C) oxidized ores of the Bushveld Complex in South Africa (6); D) a laterite above the Ora Banda Sill in western Australia (20); E) laterites above the Loma Caribe peridotite in the Dominican Republic (5); F) laterites above the Kongo–Nkamouna ultramafic massif in Cameroon (32); G) laterites in Sulawesi Island in Indonesia (13); and H) a laterite from New Caledonia (31).

This established relationship between (paleo)climate and the relative hematite and goethite abundances (24–26), coupled to the mechanistic link between iron oxide mineralogy and Pt–Pd fractionation demonstrated in this study, suggests that there may be a climatic imprint on the accumulation of Pt and Pd in ultramafic weathering zones. We explored this by examining spatial patterns in Pt/Pd ratios among distinct (paleo)climate zones (Fig. 4). Laterites in modern humid climates, where laterization remains favorable (45), consistently host Pt/Pd ratios that are either largely stable or decrease toward the surface, such as in New Caledonia, Cameroon, the Dominican Republic, and Indonesia (sites E–H in Fig. 4) (5, 13, 31, 32). These laterites in moisture-rich climates (47) also all contain abundant goethite in their upper regions (5, 13, 31, 32). While several of these laterites host a slight increase in both hematite abundance and Pt/Pd ratios at the very surface (sites F–H in Fig. 4), fractionation typically increases by <30% (5, 13, 32). A near-surface increase in hematite was included in profile 1 (humid region) of our model to replicate this trend and produces a corresponding subtle increase in Pt/Pd (Fig. 3). This mineralogical trend is also observed in other humid-region laterites (18, 48), likely reflecting slight dehydration of the uppermost part of the profile (18). In contrast, preferential Pd loss is clearly observed in soils and oxidized ores above the Stillwater Complex, the Great Dyke, and the Bushveld Complex (3, 4, 6), which are located in currently semi-arid climates (sites A–C in Fig. 4) (47).

Laterites in modern semi-arid climates may display more complex trends because current climatic conditions are generally unfavorable for their formation; thus, they reflect paleoclimatic conditions instead (45). Pt–Pd fractionation is not observed in regolith above the Ora Banda Sill in Australia because it contains abundant goethite (20), in clear contrast with the currently arid climate (site D in Fig. 4) (47). This region experienced substantially wetter conditions in the past, particularly 50–70 Ma (46), predicted to promote goethite formation; nearby goethite-rich regolith above the Murrin Murrin deposit dates to this period (49). In contrast, rainfall on the Great Dyke and Bushveld Complex has not substantially changed over the past 230 Ma (24, 46).

Observational records thus confirm that modern and/or past climates are the primary drivers of Pt–Pd fractionation and accumulation in weathering zones. The retention of PGEs within these zones is controlled by mineral-specific interfacial reactions. Variations in iron oxide mineralogy, determined by climate, create distinct depth patterns in Pt and Pd abundance. Differences in regolith mineralogy, controlled by (paleo)climate, may promote either the depletion (drier and hematite rich) or enrichment (wetter and goethite rich) of PGEs. Regional and depth variations in the degree of Pt–Pd fractionation are explained by changes in moisture-controlled iron oxide mineralogy altering the favorability of Pt and Pd retention. Ultimately, these (paleo)climate-induced trends dictate the Pt and Pd resource potential of ultramafic weathering zones.

## Materials and methods

### Mineral synthesis and characterization

Multiple batches of goethite and hematite were synthesized using modified versions of forced hydrolysis procedures (19). For goethite synthesis, 50 mL of 1 M Fe(NO_3_)_3_·9H_2_O was slowly added to 90 mL of 5 M KOH while stirring. The solution was then diluted to 1 L with ultrapure water (18.2 MΩ cm) and the pH was checked to ensure it was above 13 before aging at 70 °C for 60 h. The modified version of the hematite synthesis procedure has been previously reported (27) and is described in detail in the Supplementary Information. After aging, mineral solids were rinsed in a vacuum filtration setup (0.22 μm mixed cellulose ester [MCE] filter for goethite; 0.45 μm MCE filter for hematite) with at least 450 mL ultrapure water. The mineral solids were then resuspended and stored in aluminum foil-wrapped polypropylene bottles for later use and characterization (Table S1). Suspension concentrations were determined gravimetrically.

Hydrothermally annealed goethite was prepared using a modified version of a previously documented procedure (36). After synthesizing and washing goethite solids, the wet paste was suspended in a small amount of ultrapure water before aging in an acid digestion vessel (Parr Instrument Company) at 150 °C for 44 h (hG#1) or 1 week (hG#2). The solution was then cooled before diluting with additional ultrapure water and storing in the same manner as other mineral batches.

A subsample of each mineral batch was dried overnight in a convection oven (55 °C for goethite; 70 °C for hematite) for further characterization. X-ray diffraction patterns were collected with a Bruker d8 Advance Diffractometer (Cu Kα radiation; Fig. S2). The Brunauer–Emmett–Teller specific surface area was determined with N_2_ gas adsorption isotherms collected with a Quantachrome Nova 2000e (Table S1). Additional details regarding these measurements are provided in the Supplementary Information.

### Fluid–solid partitioning experiments

Pt and Pd adsorption to goethite, as well as Pt adsorption to hematite, were measured under three different Cl concentrations (0.5, 2, and 10 mM total Cl) at pH 4 ± 0.1. The experimental procedures followed those previously described (27); additional details regarding specific experimental conditions are provided in the Supplementary Information. A 5 mM Pd(II) stock solution, prepared by dissolving Pd-Cl in ∼50 mM HCl, and a 2.5 mM Pt(II) stock solution, prepared by dissolving sodium tetrachloroplatinate(II) hydrate in ultrapure water, were used for experiments. All stocks were prepared with reagent-grade chemicals.

In all experiments, 10 mL solutions containing up to 400 μM Pd(II) or Pt(II) were reacted with goethite or hematite for 24 h at pH 4 ± 0.10. The polypropylene tubes used were wrapped in aluminum foil to minimize photochemical reactions and were constantly mixed on end-over-end rotators. The sample pH was measured and adjusted using HNO_3_ and NaOH prior to and after adding Pd(II) or Pt(II), and at 2 and 21 h of mixing. Additional details regarding the pH electrode setup and mineral loadings used in experiments are provided in the Supplementary Information. After 24 h, the final pH was recorded, and samples were centrifuged (3,400 × *g* for 20 min) and filtered (0.22 μm MCE membrane). A portion of the filtrate was acidified to 2% HNO_3_ (trace metal grade) for Pd/Pt measurement. The remaining filtrate was later diluted with ultrapure water for measurement of Cl using a Thermo Dionex Integrion Ion Chromatograph. The Cl for each isotherm is reported as the average of measured final Cl concentrations in mineral-containing samples, ±1 SD. Pd concentrations were measured using inductively coupled plasma optical emission spectroscopy (ICP-OES) with a Thermo iCAP 7400 DUO ICP-OES in radial mode (10 ppm Sc internal standard) or with inductively coupled plasma mass spectrometry (ICP-MS) using a NexION 2000 PerkinElmer ICP-MS (20 ppb Sc internal standard). Pt concentrations were measured using ICP-OES in axial mode (1 ppm Sc) or ICP-MS (20 ppb Tb). Additional details regarding sample data processing are described in the Supplementary Information.

### Sample preparation for EXAFS spectroscopy

EXAFS spectroscopy was used to study the effects of Cl and PGE surface coverage on Pd(II) and Pt(II) coordination environments on hematite (Pt only), goethite, and annealed goethite. All samples were prepared following the same procedure as the macroscopic studies, but with larger sample sizes (50–100 mL) and mineral loadings (up to 8 g/L), in order to generate enough solids to fill sample holders. Samples that were 100 mL total volume were mixed on a shaker table. After reacting for 24 h, the solid and fluid were separated in order to pack the wet paste in a suitable sample holder; additional details regarding separation methods are provided in the Supplementary Information. Samples were then stored at −80 °C and shipped in a liquid N_2_ dry shipper to the beamline in order to prevent further reaction or desiccation. Additional information regarding the EXAFS samples is provided in Table S4.

X-ray absorption spectra of all Pd samples and select Pt samples (hG2Pt and G2lPt) were collected at beamline 4-1 at the Stanford Synchrotron Radiation Lightsource, SLAC National Accelerator Laboratory as Pd K-edge and Pt L_3_-edge fluorescence-yield data. Spectra of all other Pt samples were collected at beamline 6-BM at the National Synchrotron Light Source II, Brookhaven National Laboratory. Spectra were fit using a FEFF-based shell-by-shell method, with a structural model developed similar to prior work (27). Additional details regarding beamline setup and instrumentation, as well as the development of the structural models fit to the data are provided in the Supplementary Information.

### Pt and Pd retention and fractionation model

To assess the controls on Pt and Pd retention and fractionation, we developed a solid–fluid partitioning model that accounted for variations in goethite and hematite abundance and reactive surface area. Adsorbed Pt and Pd concentrations and Pt/Pd ratio were calculated for two simplified weathering profiles as a function of depth. The amount of adsorbed Pd and Pt to hematite and goethite was calculated using the Langmuir parameters derived from our fluid-mineral partitioning experiments (Table S2); aqueous PGE concentrations were set to 20 pM Pd and 10 pM Pt, which align with the highest concentrations measured near a PGE deposit (9) and likely represent an upper bound on dissolved concentrations in natural systems. The bulk PGE content and Pt/Pd ratio were calculated as the weighted sum of the contributions from the two mineral phases. Additional details regarding the mathematical construction of the model and justification for modeling choices are presented in the Supplementary Information.

## Supplementary Material

pgag196_Supplementary_Data

## Data Availability

Data from mineral–water partitioning experiments and EXAFS spectra for Pd(II) adsorbed to goethite and Pt(II) adsorption, as well as TEM imagery and parameters used in the laterite are available through Figshare at https://doi.org/10.6084/m9.figshare.30323788. The results from prior hematite–water partitioning experiments with Pd(II) and Pd-hematite EXAFS spectra were previously published and are available through Mendeley Data at https://doi.org/10.17632/45dzm7mgsp.2.
