# Supplementary material for: Climatic imprint on interfacially controlled platinum–palladium resources
Source: PNAS Nexus. 2026 Jun 1;5(6):pgag196. doi: 10.1093/pnasnexus/pgag196 (PMC13253563; doi:10.1093/pnasnexus/pgag196)
Supplement: pgag196_Supplementary_Data [file pgag196_supplementary_data.pdf]

PNAS nexus

Supplementary Information for

Climatic Imprint on Interfacially-Controlled Platinum-Palladium Resources

Emily G. Wright, Ivey Wang, Yihang Fang, Elaine D. Flynn, Jeffrey G. Catalano

Corresponding author: Jeffrey G. Catalano
Email: catalano@wustl.edu

This PDF file includes:

Supplementary text
Figures S1 to S22
Tables S1 to S11
SI References

Supplementary Information Text

Extended Materials and Methods

Mineral Synthesis and Characterization. Goethite and hematite synthesis procedures were modified from previously published methods (1). The goethite synthesis method is described in the main text. The hematite synthesis method follows our previous work (2). For hematite synthesis, 300 mL of 1 M NaOH and then 50 mL of 1 M $NaHCO_3$ were slowly added to a 500 mL continuously stirred solution of 0.2 M $Fe(NO_3)_3 \cdot 9H_2O$. The hematite synthesis solution was then aged at 98°C for 5 days, then washed with at least 450 mL ultrapure water (18.2 MΩ cm) in a vacuum filtration setup (0.45 μm MCE filter) before resuspending and storing.

The surface area and mineralogy of synthesized mineral batches were determined. Most X-ray diffraction (XRD) patterns were collected with the following conditions: 10-90° 2θ, 0.02° step size, and 0.2 second count time. The XRD pattern for G#3 was collected over the range of 15-65° 2θ. The XRD patterns for G#5 and H#2 were collected with the following conditions: 13-65° 2θ, 0.02° step size, and 0.5 second count time. The Brunauer-Emmett-Teller (BET) specific surface area was measured using N_2 gas adsorption isotherms only for mineral batches used for adsorption isotherm experiments. Prior to BET analysis, the dried, unground mineral was degassed under vacuum at either room temperature (goethite) or 100°C (hematite) for at least 18 hours.

Fluid-Solid Partitioning Experiments. Platinum and Pd adsorption to goethite, as well as Pt adsorption to hematite, were measured under a range of chloride (0.5, 2, and 10 mM) concentrations at pH 4±0.1. The chloride concentrations investigated were chosen to be both consistent with the range of chloride concentrations measured in relevant tailings porewater and groundwater near a PGE deposit (3-6), as well as to align with previous Pd(II) adsorption experiments performed with hematite and 2-line ferrihydrite (2). Substantially elevated Pd and Pt, much higher than likely natural concentrations (7-9), were required for the experiments in order to ensure dissolved Pd or Pt remained quantifiable by available instrumentation. Thus, experiments were performed at pH 4 to ensure Pd(II) solubility and to still fall within the expected range of pH values in relevant weathering environments (10-13).

For use in the experiments, the 5 mM Pd(II) stock and 2.5 mM Pt(II) stock were prepared as described in the main text. For the 0.5 mM chloride experiment with Pd(II), a portion of the Pd(II) stock was diluted to 1 mM Pd(II) with ultrapure water. Additional supplemental samples at low Pt(II) concentrations for the 0.5 and 2 mM Cl goethite isotherms were prepared with a 0.5 mM Pt(II) stock prepared by further diluting the 2.5 mM Pt(II) stock with ultrapure water.

Constant chloride concentrations in experimental samples were maintained through the addition of dissolved NaCl (10 and 100 mM stock solutions), accounting for the chloride contribution from the Pd(II) or Pt(II) stock solutions. Mineral-free controls and physical triplicate samples were also prepared. The Pd(II) experiment with goethite in 10 mM total chloride used mineral loadings of both 1 and 4 g/L; the equivalent Pt(II) experiments used a mineral loading of 1 or 2 g/L, for goethite and hematite respectively. The experiments in 2 mM total chloride were prepared using a mineral loading of 1 g/L (Pd; Pt with hematite) and/or 0.5 g/L (Pd; Pt with goethite). The experiments in 0.5 mM total chloride used a mineral loading of 0.5 g/L (Pt with hematite), 0.4 g/L (Pd), or 0.25 g/L (Pt with goethite). After initial data analysis, additional low Pt(II) concentration samples for all chloride conditions with goethite were prepared in order to evaluate differences at low surface coverage; the same mineral loadings were used, but the mineral batch differed (see **Table S1**). Additional experiments following the same procedures were used to evaluated Pd or Pt adsorption to hydrothermally-annealed goethite (0.5 g/L) in 2 mM total chloride; Pd(II) adsorption isotherms were collected with both annealed goethite batches (hG#1 and hG#2), while Pt(II) adsorption was only studied on the goethite annealed for 1 week (hG#2). The effect of ionic strength was also studied by collecting an additional Pt(II) adsorption isotherm with hematite (1 g/L) in the presence of 2 mM total chloride and 8 mM $NaNO_3$.

As previously described (2), the standard pH electrode used (filled with a commercial 3.5 M KCl and AgCl solution) leaked chloride into samples via the porous junction. At low total chloride concentrations, this effect was substantial. The Pd(II) experiment with goethite in 10 mM total chloride and a portion of the data for Pd(II) adsorbed to goethite (0.5 g/L) in 2 mM total chloride were measured with this initial pH electrode setup. All other experiments were conducted with a chloride-free pH electrode setup, where the outer chamber of a double-junction electrode was replaced with 3 M KNO_3 and the electrode was stored in 3 M KNO_3.

Adsorbed Pd and Pt were calculated by difference, normalized to the available surface area. The mineral-free controls were used to correct for various losses; a linear regression was applied between the target starting concentrations and actual measured Pd or Pt in these samples, which was then used to calculate the initial Pd or Pt in all experimental samples. Error was calculated by adding in quadrature the average error for the weighted calibration curve, the relative standard deviation from physical triplicate samples, and the sample relative standard deviation from triplicate instrumental measurements. Samples were precipitation was suspected were removed from further data analysis. Physical triplicate samples that had drifted outside of the acceptable pH range (4±0.1) were used to calculate error but were otherwise removed from analysis. Two-component, or dual, Langmuir adsorption isotherms were fit to each dataset with a non-linear least squares method (Levenberg-Marquardt algorithm).

Thermodynamic Modeling of Pt and Pd Speciation. Existing thermodynamic data indicates that Cl^- and OH^- are the dominant inorganic ligands which complex Pd and Pt under oxidizing and weakly acidic conditions (14). While there is some prior literature suggesting that organic molecules also complex Pt and Pt, the data are too limited to include in speciation models (15-17) and beyond the scope of this current work. No widely-used thermodynamic database includes data for Pt species (18-22). Additionally, there is considerable disagreement about the interpretation of experimental results (23-25). Some of the more comprehensive surveys of the stability of aqueous Pt(II) species (23, 26-28) result in drastically different predictions of Pt(II) speciation under conditions relevant to the experimental system (**Fig. S1**). The (29)greatest disagreement is regarding the stability of Pt(II)-OH species, where calculated equilibrium constants can vary by up to 10 orders of magnitude (23). However, there are also multiple orders of magnitude disagreement for Pt(II)-Cl complexes (23). This considerable disagreement makes it challenging to predict Pt(II) speciation with any degree of confidence under the experimental conditions tested.

In comparison to Pt, predictions of Pd aqueous speciation suggest that Pd-Cl complexes are likely more important (**Fig. S1**). However, there still is disagreement regarding the stability of Pd complexes (29-31), hindering our ability to make quantitative predictions (**Fig. S1**). Similar to Pt, the greatest disagreement is in the stability of Pd-OH species (**Fig. S1**). For example, while several studies present equilibrium constants for $Pd(OH)^+$ (30, 31), a reanalysis of prior thermodynamic data could not confirm its existence (29). Similarly, this reanalysis was unable to fully constrain the equilibrium constant for $Pd(OH)_{2(aq)}$ and was only able to provide an upper bound on its stability (29). While there is greater agreement on the stability of Pd-Cl species (29, 31), continuing disagreement in the stability of Pd-OH species results in predictions of the importance of Pd-Cl species varying by several orders of magnitude (**Fig. S1**).

It is thus not currently possible to quantitatively compared Pd and Pt speciation under the experimental conditions studied because of the substantial uncertainty in the stability of major PGE species. In the case of Pd, the speciation models presented in **Figure S1D** likely represent a maximum bound on the importance of $Pd(OH)_{2(aq)}$ and minimum bound on the relative abundances of Pd-Cl species. The relative strength of Pt-Cl complexation is poorly constrained (**Fig. S1**), although these existing datasets suggest that such complexation may be weaker compared to Pd, implying aqueous speciation under the conditions assessed may differ substantially between Pd and Pt.

Surface Charge. In order to assess the effect of chloride on the surface charge of goethite, the zeta potential of this mineral was measured in the presence of varying amounts of NaCl and compared with prior hematite data (2). Samples consisting of 50 mL of 1 g/L goethite with 0.5, 2, and 10 mM NaCl at pH 4±0.1 were prepared, following the same general procedures as the macroscopic adsorption experiments. None of these samples contained any Pt(II) or Pd(II). Samples were measured in triplicate using a Malvern ZEN3600 Zetasizer.

Sample Preparation for EXAFS Spectroscopy. In order to collect the solids from large-volume reactions after 24 hours for later analysis, the samples were separated and packed four different ways. Initial Pd samples were transferred to round-bottom tubes and centrifuged at high speed (7099 g) for at least 60 minutes. The supernatant was then entirely decanted and the solid phase was scraped from the bottom of the tube and loaded into a Delrin sample holder and sealed with Kapton tape (sample prep method 1). For all other samples, the samples were centrifuged at lower speed (minimum 3398 g for at least 45 minutes) in 50 mL conical tubes before decanting the majority of the supernatant. For goethite

and hydrothermally-annealed goethite samples, the solids were then resuspended in the remaining supernatant to create a concentrated suspension. This suspension was filtered using a reusable syringe setup in order to collect the solid onto a 0.22 μm MCE filter membrane. This filter membrane was then either sealed with Kapton film and Kapton tape (sample prep method 2) or the solid was scraped off the filter membrane and packed in either a Delrin (all Pd samples, hG2Pt, G2lPt) or aluminum (all other Pt samples) sample holder and sealed with Kapton tape (sample prep method 3). For hematite samples, after the initial centrifugation, all of the supernatant was removed and the solid phase was scraped from the bottom of the tube and packed into an aluminum sample holder, sealed with Kapton tape (sample prep method 4). For all sample preparation methods, only plastic tools were used to prevent the potential reduction of Pd(II)/Pt(II). The remaining supernatant was filtered with a 0.22 μm MCE syringe filter. A portion was reserved for further dilution and measurement of chloride; the remaining was acidified and later analyzed for total dissolved Pd or Pt.

Palladium K-edge and select Pt (hG2Pt, G2lPt) L_3-edge X-ray absorption spectra were collected at beamline 4-1 at the Stanford Synchrotron Radiation Lightsource. A cryogenically-cooled Si (220) double-crystal monochromator was used to scan the incident beam energy; in order to minimize harmonic contributions, the second crystal was detuned by 25% for Pd and 30% for Pt. A 32-element solid-state Ge energy-dispersive detector was used to collected fluorescence-yield data. In order to prevent beam-induced reduction, samples were measured at 77 K in a liquid N_2 cryostat. Multiple scans were averaged and corrected for deadtime using the SixPACK interface to IFEFFIT (32, 33).

All other remaining Pt L_3-edge spectra were collected at beamline 6-BM at the National Synchrotron Light Source II. At this beamline, a cryogenically-cooled Si (111) double-crystal monochromator, followed by a flat harmonic rejection mirror, was used to scan the incident beam energy. Fluorescence-yield data were collected with a 7-element Si drift detector. Samples were kept at approximately 78 K using a Linkam cold stage. Multiple scans from NSLS-II were averaged using Athena (34).

The backscattering phase and amplitude functions used for fitting were generated in FEFF8L (35) with Larch (36). For analysis of Pd EXAFS spectra, a modified version of the Pd_2OCl_2 (37), where Fe atoms replaced two Pd atoms, was used. For analysis of Pt EXAFS spectra, a modified version of the crystal structure of *trans*-$[PtCl(OH)(NH_3)_2]\cdot H_2O$ (38), where an Fe atom replaced one Pt neighbor at 3.328 Å, was used. SixPACK was then used to refine the structural models to the EXAFS spectra. For Pd, an amplitude reduction factor (S_0^2) of 0.87, based on fitting a spectrum of Pd foil, was used. For Pt, S_0^2 was set to 1.0; preliminary fitting of a spectrum of Pt foil returned a value of S_0^2 of 0.94±0.06, so 1.0 was chosen given the relatively large uncertainty in the value. A structural model for both Pt and Pd was developed similar to prior work (2), where the coordination number (CN) for the two paths in the first shell (Pt/Pd-Cl and Pt/Pd-O) was set to be equal to 4, assuming a square planar coordination. In order to reduce parameter correlations, the σ^2 for the first shell paths was set to be equal. The FEFF calculations for both Pt and Pd identified several multiple scattering paths involving O or Cl: Pd-Cl-Cl'-Pd, Pd-Cl-Pd-Cl'-Pd, and Pd-Cl-Pd-Cl-Pd scattering paths, or their Pt and/or O equivalents, where Cl'/O' indicate a Cl or O backscatterer distinct from the initial anion. For these multiple scattering paths, the CNs were set equal to the CN for the associated Pt/Pd-O/Cl single paths, with interatomic distances and σ^2 values set as double those of the corresponding single scattering paths. The addition of these multiple scattering paths introduced no new fitting parameters. Additionally, Pt/Pd-Fe paths were added, with a single σ^2 used for all of these second shell Fe neighbors. Based on preliminary fitting of Pd samples with the largest second shell feature, σ^2 was set to be equal to 0.002 for all samples. A single ΔE_0 was fit for all paths. Palladium spectra were fit in R-space from 1.1 to 4.5 Å using the Fourier transform *k*-range of 3.0 to 13.5 $Å^{-1}$. Platinum spectra were fit over the same R-space range as Pd data, but using the Fourier transform *k*-range of 3.0 to 12.3 $Å^{-1}$. These structural model fits were compared with previous published data of Pd(II) adsorbed to hematite (2); note that second shell values provided for comparison (**Table S11**) are from model C reported in the supplementary material, which was the same fitting approach as taken in this current work.

TEM Imaging. In order to rule out the formation of Pd(II) precipitates, which could explain a strong second shell feature observed with EXAFS spectroscopy in some samples, scanning transmission electron microscopy (STEM) energy dispersive X-ray spectroscopy (EDS) element mapping and high-angle annular dark-field (HAADF) images were used to observe the spatial distribution of Pd associated with goethite. A sample containing 0.25 g/L goethite, 43 μM Pd, and approximately 2 mM total chloride

was prepared following the same general procedure as other adsorption samples in order to create a sample with approximately the same amount of adsorbed Pd as EXAFS sample G2h (**Table S4**), which had the strongest second shell feature (**Fig. S13**). After 24 hours reacting, a 0.5 mL subsample was centrifuged at 11395 g for seven minutes. The solution was then decanted and the tube was refilled with ultrapure water before repeating the centrifugation procedure. This procedure was repeated again before resuspending the goethite particles in ethanol. The sample was then ultrasonicated for five minutes before a droplet was placed on a copper lacey carbon transmission electron microscopy (TEM) grid and air dried.

Bright-field (BF) images under TEM mode were also used to investigate the hydrothermally-annealed goethite synthesized. A drop of the annealed goethite suspension was suspended in ethanol for dilution. The goethite in ethanol solution was then ultrasonicated for five minutes to better disperse the sample and minimize aggregation. A drop of this dispersed solution was then transferred onto a copper lacey carbon grid and air dried.

STEM and TEM data were collected using a JEOL JEM-2100F Field Emission Scanning Transmission Electron Microscope. This instrument was operated at a 200 kV acceleration voltage. A larger spot size (4 nm) was used during the TEM image acquisition to minimize beam damage.

Pt and Pd Retention and Fractionation Model. For each of the three chloride concentrations, the amount adsorbed *A* (in µmol/g) of element *Z* to a given mineral *M* is calculated as a function of depth *d* using the following equation:

$$A_{Z,M}(d) = SS_M(d) \times (\Gamma_{1,Z,M} \times \frac{K_{1,Z,M} \times C_z}{1 + K_{1,Z,M} \times C_Z} + \Gamma_{2,Z,M} \times \frac{K_{2,Z,M} \times C_Z}{1 + K_{2,Z,M} \times C_Z})$$

where *SS* is the mineral-specific surface area (in m^2/g), *C* is the aqueous concentration (in µM; Pd was set to 20 pM and Pt to 10 pM), Γ is the maximum binding capacity (in µmol/m^2), and *K* is the adsorption affinity (in µM^{-1}). The dual Langmuir constants fit to the experimental data (**Table S2**) were used for this model. For modeling Pd adsorbed to hematite, dual Langmuir isotherms were refit to previous data (2) in order to improve fits at low concentrations and maintain better consistency with the current fitting approach (**Table S2**). In order to calculate the total concentration of PGEs associated with iron (oxyhydr)oxides, the following equation was used to calculate the weighted sum of the contributions from each mineral phase:

$$Z(d) = \left(A_{Z,H}(d) \times R(d) + A_{Z,G}(d) \times (1 - R(d))\right) \times 1000 \times M_Z$$

where the concentration of element *Z* (in ng/g) as a function of depth is calculated as a function of the amount adsorbed to hematite (*H*) and goethite (*G*), weighted by the ratio *R* (Hematite/Hematite+Goethite), and *M* is the molar mass (in g/mol). The concentrations of Pd and Pt were then also used to calculate the Pt/Pd ratio as a function of depth. Note that inconsistent thermodynamic data for Pt(**II**) (**Fig. S1**) precludes the development of surface complexation or reactive transport models to study Pt and Pd retention within these zones, necessitating our specific modeling approach.

Laterites commonly contain a mixture of goethite and hematite and their abundance frequently varies with depth. Prior observations informed the development of two models for iron (oxyhydr)oxide mineralogy in laterites as a function of depth. Some prior studies of laterites have determined quantitative mineralogy with depth, where the Hematite/Hematite+Goethite ratio ranges from 0 to 1 (39-44). These lateritic profiles were used as the basis for the synthetic profiles used in the Pt and Pd retention and fractionation model. Profile 1 models a scenario of yellowing, where goethite content increases towards the top and/or replaces hematite higher up in the profile (**Fig. 3**). This may occur in more humid and saturated profiles, like in the Amazon (e.g., 42, 45, 46). Profile 2 models reddening, where the hematite content increases at the expense of goethite closer to the top of the profile (**Fig. 3**). Reddening likely reflects dehydration of the uppermost part of the profile, and has been documented in numerous laterites, such as in New Caledonia, Australia, and Indonesia (e.g., 47, 48, 49).

Within laterites, the iron (oxyhydr)oxide abundance can range from 30-90% of the total mineral assemblage (e.g., 39, 40, 43, 44). While this model implicitly treats the entire laterite as a mixture of goethite and hematite, other minerals present in the laterite could further alter the Pd and Pt retention trends. However, hematite and goethite are likely to be the major controls on Pt and Pd behavior due to their ubiquitous abundance across laterites (39-44, 50) and the high affinity of Pd and Pt for iron (oxyhydr)oxides observed in the present study (**Fig. 1**) and previous analyses of natural samples (e.g.,

50, 51). For example, kaolinite is variably abundant in some, but not all, laterites (e.g., 39, 43, 44, 50), suggesting that it cannot be universally responsible for Pt and Pd behavior. Additionally, a prior adsorption experiment demonstrated that Pt has a much lower affinity for this phase compared to hematite and goethite under similar conditions to this current study (52). Inversely, Pd and Pt are known to also be associated with Mn (oxyhydr)oxides (51), but the far lower abundance of these phases (40, 41, 43, 53) implies that they cannot be the dominant adsorbent phase in laterites. Finally, while other iron (oxyhydr)oxides, such as magnetite and maghemite, may occasionally be present in laterites, they are substantially lower in abundance compared to hematite and goethite (e.g., 43, 44), indicating that they are less important phases. Additionally, our previous study (2) demonstrated that Pd has essentially the same adsorption affinity for ferrihydrite and hematite, suggesting that PGEs will not have a unique affinity for all iron (oxyhydr)oxide minerals. Prior sequential extractions of natural samples also indicated that Pt and Pd were associated specifically with hematite and goethite, respectively (50). These observations suggest that this model involving only hematite and goethite should broadly reflect Pt and Pd behavior in laterites, even if additional mineralogical complexity may have a minor effect.

In addition to these mineralogical trends, particle size/surface area effects may in turn alter the amount of PGE retention. Previous Pd(II) work with ferrihydrite (2) and the current work with annealed goethite (**Fig. S20**) strongly suggest that the degree of crystallinity does not substantially alter the adsorption affinity. However, coarsening effects could alter the relative balance of reactive hematite versus goethite as a function of depth in these weathering zones; for adsorption, the ratio of hematite versus goethite surface area should control the amount of retention and the degree of platinum-palladium fractionation, not the total bulk hematite or goethite content.

There is very limited prior work on the surface areas of natural hematite and goethite particles within these weathering zones, with substantial caveats. The crystallite size of goethite frequently becomes larger towards the surface, reflecting aging and recrystallization (48, 54). Thus, for both depth profiles, a scenario of goethite coarsening toward the surface was modeled (**Fig. 3**). Data on hematite in these weathering zones is even more limited, but investigators generally suggest that the hematite crystallite size is larger than goethite (55-57). Hematite crystallite sizes trends with depth are unclear: authors have reported no change with depth, increasing size towards the surface, and decreasing size towards the surface (39, 46). In the model, hematite surface area is treated as constant throughout the weathering profile (**Fig. 3**).

However, it should be noted that estimates of surface area based on crystallite size are likely inaccurate because they assume an ideal crystallite shape and do not account for additional porosity (58). For instance, the annealing procedure with goethite resulted in reduced surface area (**Table S1**), but did not drastically change the observed particle size (**Fig. S19**). Alternatively, surface area estimates of goethite based on crystallite size are also likely to be substantially overestimated because goethite particles commonly occur as “bundles” of rods and the measured mean coherent domain (by XRD) does not reflect the true size of these multi-domainic goethite particles (58, 59). Finally, the presence of poorly crystalline phases may not be well represented in XRD patterns collected from natural samples (60), which could lead to underestimation of their abundance. Hematite and goethite have both been occasionally found to have low crystallinity in laterites (59, 61-63). Small, poorly crystalline phases could represent a major source of reactive surface area, but may not be well documented by the existing literature.

Several prior studied laterites contain platinum group minerals (PGMs) (53, 64, 65). The presence of these grains may lead to locally elevated PGE concentrations and alter the Pt/Pd ratio in portions of the weathering profile (53). Elsewhere, Pt and/or Pd concentrations are below the detection limit in some regions (66), which makes it difficult to identify fractionation and depth-dependent trends.

Additionally, PGE retention is modeled based on adsorption experiments at pH 4, when surface complexation is pH-dependent. Generally, pH increases with depth within these weathering zones (13). A decrease in pH results in less adsorption of Pd to hematite (2), which might indicate that Pt and Pd may adsorb in greater amounts with increasing pH.

An additional limitation is that, to our knowledge, no prior work that has quantified PGE concentrations as a function of depth in laterites has also collected quantitative mineralogical information. At best, a qualitative ranking of mineralogy is provided (13, 53, 67). Thus, prior investigations lack all of the information that would be necessary to evaluate the predictions from the model.

Supplementary Results

Adsorption Isotherm Experiments. All adsorption datasets were generally well fit with an unweighted dual Langmuir isotherm (**Fig. 1**). Initial fits to the 0.5 mM total chloride experiment for goethite with Pd(II) struggled due to poor constraints on the maximum binding capacity. Due to the limited solubility of Pd(II), particularly in chloride-poor fluids, it was not possible to obtain adsorption values for higher Pd(II) concentrations. Since there is no clear means through which the adsorption capacity would substantially shift at the same pH in the presence of varying amounts of chloride, the lower binding capacity for the 0.5 mM chloride experiment with goethite and Pd(II) was fixed to be the same as the 2 mM chloride experiment (**Table S2**). Prior data on Pd(II) adsorbed to hematite (2) were also refit with dual Langmuir isotherms in order to qualitatively improve the fit and maintain consistency for better comparison of datasets. Constraints were similarly necessary in order to fit Pd(II) adsorption in 0.5 mM chloride and both binding capacities were fixed to be identical to the 10 mM chloride experiment (**Table S2**).

Additional samples of Pt(II) adsorbed to goethite were prepared to better evaluate trends at low Pt(II) concentrations. However, it was not possible for us to prepare these samples using the same batches of goethite that were used to generate the original isotherms. While the low Pt(II) data aligns well with the prior 0.5 and 2 mM Cl isotherms, there is a discrepancy with the previous 10 mM Cl dataset (**Fig. S22**). This difference could be due to slight differences in particle morphology between batches that altered the total binding capacity, which is supported by prior work (68). Thus, in order to fit dual Langmuir isotherms, both portions of the isotherm were simultaneously fit, with an additional scaling value that describes the differences between datasets. By convention, a scaling value of 1 indicates that the higher Pt coverage experiment is in perfect alignment with the lower Pt coverage experiment. For plotting the isotherms, the measured adsorption for the higher Pt portion of the isotherm was divided by the scaling factor. This approach fits all of the experimental data (**Fig. S22**). Notably, the scaling factor obtained is the same for the 0.5 and 2 mM Cl isotherms (**Table S2**), which were constructed using the same batches of goethite (**Table S1**).

Surface Charge. The zeta potential of goethite was measured to assess whether its charging behavior differed as a function of chloride. For comparison, prior work found that the surface charge of hematite and 2-line ferrihydrite remained relatively constant (and positive) across the range of chloride concentrations investigated at pH 4±0.1 (2). For goethite, the measured zeta potentials were all positive, indicating a positive surface charge (**Table S3**). Additionally, all measured zeta potentials were within two standard deviations, suggesting that surface charge is relatively constant despite changing concentrations (**Table S3**).

EXAFS Data Analysis of Pt(II) Samples. EXAFS spectroscopy was used to evaluate the effect of aqueous chloride concentration and Pt(II) surface coverage to hematite and goethite on Pt(II) binding configuration. The effect of hydrothermally annealing goethite was also briefly evaluated in order to test the possible effect of Fe vacancies on Pt(II) coordination environment. The specific preparation details for each EXAFS sample are presented in **Table S4**.

All spectra are dominated by a large feature between ~1.3 and ~2.3 Å (R+ΔR) in the Fourier transform, with some additional features between ~2.6 and ~4.0 Å (R+ΔR) (**Fig. S20**). There are consistent variations in the Fourier transform magnitude of hematite spectra with changing chloride concentration and Pt(II) surface coverage. The goethite spectra are largely similar to the hematite spectra in the first shell region, but differ slightly at high *k* and in the second shell region (**Fig. S6**). Additionally, all goethite spectra are nearly identical despite changing experimental conditions (**Fig. S6**). Notably, principal component analysis (PCA) of the goethite spectra suggests that more than 98% of the total variance can be explained by the first component, with the indicator (IND) value also minimizing on this component (**Fig. S7**; **Table S5**). In contrast, PCA of the hematite spectra indicates that the majority of spectral variance is described by the first two components (**Table S6**), which adequately reconstruct all spectra (**Fig. S8**).

The structural model fits reproduce these data (**Fig. S6**). The first shell feature was well fit with a mixture of O and Cl neighbors at ~2.0 and ~2.3 Å, consistent with distances obtained in previous work (38, 69, 70). For hematite, the fits yield between 1.7 and 2.5 O neighbors and between 1.5 and 2.3 Cl neighbors (**Table S7**). For the goethite samples, where there is considerably less variability in the EXAFS spectra (**Fig. S6**), between 2.2 and 2.4 O neighbors and 1.6 and 1.8 Cl neighbors (**Table S8**) are obtained by the fits. In order to accurately capture additional features in the Fourier transform in all spectra, multiple scattering and three Pt-Fe paths at ~3.06, ~3.69, and ~3.89 Å were added. The σ^2 for

these Pt-Fe paths was constrained to 0.002 on the basis that an inner-sphere Pt species should be fairly rigid (supported by the low σ^2 fit for the first shell paths; **Tables S7**,**S8**). For both hematite and goethite, small CNs for the shortest Pt-Fe path are obtained (within error of 0 to 0.3; **Tables S7**,**S8**); however, this path was required in order to adequately constrain the first shell fit due to interference. In contrast, relatively large CNs for the two longer Pt-Fe paths are obtained (1.0-1.6 for Fe2; 0.7-1.2 for Fe3; **Tables S7**,**S8**). Generally, the CNs for Fe1 are largest for the hematite samples, while goethite samples have generally larger CNs for Fe2 and Fe3, although these differences are within error (**Fig. S11**). There are no clear trends between the fit of the first shell and any of the Fe neighbors (**Fig. S11**).

The fit results (**Tables S7**,**S8**) are consistent with inner-sphere Pt-Cl ternary surface complexation on both hematite and goethite. The high total Fe CNs (**Fig. 2**; **Tables S7**,**S8**) suggest that Pt(**II**) binds to both minerals only as inner-sphere species, with no evidence for an outer-sphere component. On both minerals, the short Pt-Fe distance of ~3.06 Å would be consistent with a minor edge-sharing bidentate species, such as what was previously identified for Pd(**II**) adsorption on hematite (2). However, at least one species with multiple Fe neighbors at longer Pt-Fe distances is required to form on both minerals in order to explain the high total Fe CNs. For both minerals, there are multiple possible geometries that would satisfy this requirement. However, it is not possible to definitively confirm which specific binding geometries are actually present in the EXAFS dataset and the large number of possible configurations (including variations in protonation and hydrogen bonding) make computational approaches impractical. Despite this, the general trend suggests that Pt binds to goethite and hematite in a similar manner and that Pt prefers corner-sharing over edge-sharing geometries, the opposite of Pd.

For goethite, there are at least two possible binding geometries on the {101} surface: a monodentate complex and a bridging bidentate complex (**Fig. S9**). On the goethite {210} surface, there are several different options that would be consistent with at least one of the two Fe distances fit, including possibilities that would result in greater than two Fe neighbors (**Fig. S9**). Notably, a bridging bidentate complex on edge-sharing octahedra yields one Fe neighbor at ~3.62 Å and one neighbor at ~3.83 Å, due to asymmetry in the Fe-O bond length in the distorted iron octahedra (**Fig. S9**), which would be broadly consistent with the two long Fe distances fit. Note that all of these distances assume no relaxations at the surface and that the true distances for these geometries are likely different.

On hematite, there are also multiple possible geometries that would be broadly consistent with the fit results (**Fig. S10**). On the {012} surface, bridging bidentate complexes can occur on both possible terminations (**Fig. S10**). There are also additional possible configurations on the {110} surface, including at least two possible geometries where there are three Fe neighbors (**Fig. S10**).

EXAFS Data Analysis of Pd(II) Adsorbed to Goethite. EXAFS spectroscopy was used to investigate Pd(**II**) binding mechanisms to goethite using a suite of samples. The effects of aqueous chloride concentration and Pd(**II**) surface coverage on the coordination environment of adsorbed Pd(**II**) were evaluated. After some initial Pd EXAFS spectra displayed a unique, strong second shell feature (**Fig. S13**), the effect of annealing goethite was also investigated in order to study the possible influence of surface vacancies. The specific sample preparation details are provided in **Table S4**.

The spectra of Pd(**II**) adsorbed to goethite and hydrothermally-annealed goethite are dominated by two overlapping features between ~1.5 and ~1.8 Å (R+ΔR) in the Fourier transform (**Fig. S13**), which vary in relative magnitude as a function of both the aqueous chloride concentration and Pd(**II**) surface coverage. These features were fitted with O and Cl neighbors at ~2.0 and ~2.3 Å, respectively, consistent with prior work (2, 71, 72) At lower chloride concentrations, the Cl component decreases (**Fig. S13**), which is qualitatively consistent with the prior work on Pd(**II**) adsorbed to hematite and ferrihydrite in similar fluids (2). However, there are also now distinct trends in the O and Cl components based on the Pd(**II**) surface coverage; the Cl feature is consistently more prominent at lower surface coverages (**Fig. S13**). The primary mode of variation between spectra are dampening of oscillations in the EXAFS spectra at high *k* (**Fig. S13**). All spectra share isosbestic points and principal component analysis (PCA) indicates that the majority of the spectral variance can be described by two components (**Table S9**). All spectra are well reconstructed with just the first two components (**Fig. S12**). These findings support the presence of at least two distinct end-member species.

An additional feature between ~2.2 and ~3.2 Å (R+ΔR) is present in all spectra except for G10lPd (**Fig. S13**). During initial fitting this feature was not fully reproduced by the addition of just multiple scattering; based on wavelet transforms of select spectra (**Fig. S14**), this feature is distinct from multiple scattering and results from contributions at high *k*, indicative of a heavy element (Fe or Pd). If this feature

was produced by Pd neighbors, this would be consistent with the formation of a Pd precipitate or a polynuclear species. However, the isotherm behavior is not consistent with precipitation occurring (73). Additionally, PdO and hydrous PdO could not be reconstructed using the first two components of the PCA (**Fig. S17**), which further rules out a nanoscale precipitate or Pd hydroxide polymers/clusters. Additionally, element mapping of goethite solids reacted with Pd using STEM demonstrates that Pd is broadly dispersed across the goethite surface, with no evidence for precipitation (**Fig. S18**). Thus, multiple lines of evidence suggest that the heavy element cannot be Pd, and thus must be Fe. In order to reproduce this feature, the structural model included multiple scattering and three distinct Pd-Fe paths at ~2.93, ~3.17, and ~3.6 Å (**Table S10**). Only a single Pd-Fe path at 3.62 Å was used for fitting G10lPd, which did not contain this feature in the Fourier or wavelet transforms (**Fig. S13**,**S14**).

The Pd structural model fits reproduce the data well (**Fig. S13**) and indicate between 1.1 and 2.1 Cl neighbors and between 1.9 and 2.9 O neighbors (**Table S10**), with larger Cl CNs generally observed at higher aqueous chloride concentrations. Consistent with the qualitative differences observed in the Fourier transform magnitudes (**Fig. S13**), smaller Cl CNs and greater Fe CNs are fit at higher surface coverages (**Table S10**). Based on the systematic trends observed in the fits, the dataset indicates the presence of at least two species with differing affinities, which is consistent with the PCA results (**Fig. S12**). There is a weak negative correlation (R^2 = 0.49) between the O CN and the CN for the furthest Fe neighbor (**Fig. S15**) and a strong positive correlation between the O CN and the two shorter Fe CNs (R^2 = 0.67 for Fe4; R^2 = 0.87 for Fe5; **Fig. S15**). There is also a positive correlation between the O CN and the sum of all Fe paths (R^2 = 0.76; **Fig. S15**), although the correlation is slightly better when comparing O CN and the sum of all edge-sharing Pd-Fe paths (Fe4 and Fe5; R^2 = 0.81; **Fig. S15**). There is also a very strong positive correlation between the number of Fe4 and Fe5 neighbors fit (R^2 = 0.91; **Fig. S15**).

These trends indicate a Pd-Cl species with a Pd-Fe distance of ~3.6 Å, favored at low surface coverages and high chloride concentrations. This species has a high affinity for goethite and could be the potential monodentate $PdCl_3$ species proposed to form on hematite and ferrihydrite with the same approximate Pd-Fe distance (**Table S11**) (2). Alternatively, this species could have a different geometry; if it contains only at most two Cl ligands, there are many possible corner-sharing bidentate species that would yield Fe distances consistent with the fits (**Fig. S9**). The low Fe CN (0.5; **Table S10**) for G10lPd, where shorter Fe distances were not quantified, may indicate the presence of an additional outer-sphere component.

At high Pd(II) concentrations and lower chloride concentrations, the fits indicate a lower affinity species that contains few or no Cl ligands which is strongly associated with the shorter Pd-Fe paths; the high chloride concentration and low Pd(II) surface coverage of G10lPd may have suppressed its formation. However, the resulting Fe CNs from the fits are incompatible with traditional inner-sphere adsorption geometries. The shorter Pd-Fe distances (2.93-2.94 and 3.16-3.18 Å; **Table S10**) should correspond to edge-sharing geometries. These geometries are only associated with one Fe neighbor typically. However, the sum of all Fe CNs is greater than and not within error of 1, excluding G10lPd (**Fig. 3**; **Table S10**). While the assumption that these Pd species are relatively rigid (σ^2 = 0.002) does affect the CNs fit for these Pd-Fe paths, a larger σ^2 would result in even larger Fe CNs. Attempts to model an adsorbed Pd atom with two or more close Fe neighbors were unsuccessful. However, an alternative model where Pd replaces an Fe site in a structural chain yields two Fe neighbors at ~3.02 Å (**Fig. S16**), which would be more broadly consistent with the structural model fits. This result indicates that Pd may become entrapped within the goethite structure, a unique binding mechanism not previously observed on hematite or ferrihydrite (2).

Element Mapping of Goethite Particles Reacted with Pd(II). In order to evaluate the interpretation that the second shell feature in the Pd EXAFS spectra (**Fig. S13**,**S14**) was caused by Fe neighbors, rather than Pd, a sample of Pd adsorbed to goethite was prepared and imaged with STEM. This sample was modeled to be similar to G2hPd, which had the most prominent second shell feature in the Fourier transform EXAFS spectra (**Fig. S13**). Element mapping indicates that, despite the low signal level, Pd is homogeneously dispersed across the sample and clearly associated with the Fe element map and goethite particles (**Fig. S18**). The uniform distribution of the Pd element map (**Fig. S18**) is consistent with adsorption, not precipitation, supporting the interpretation that the second shell feature is due to Fe, not Pd.

Effect of Annealing Goethite on Pd(II) and Pt(II) Adsorption. We initially hypothesized that the unique behavior of Pd(II) on goethite was driven by defects on the goethite surface. Defects, which are common in natural and synthesized goethite (74, 75), are known to play an important role in controlling the reactivity and bulk and surface properties of goethite (74, 76). In particular, prior work has shown that Fe(II)-induced recrystallization of goethite, a process that involves adsorption of Fe(II), is driven by surface defects (77). To test this hypothesis, Pd(II) and Pt(II) adsorption to hydrothermally-annealed goethite was evaluated. Annealing goethite has been previously shown to result in a lower surface area and fewer Fe vacancies and a higher Fe/O ratio near the surface (77). After annealing for 44 hours, the goethite had a slightly lower surface area (21.2 m^2/g; **Table S1**). Annealing for a longer period (1 week) did not substantially change the surface area (23.6 m^2/g; **Table S1**). TEM imaging demonstrates that annealed goethite needles have slightly more coherent and clean terminations (**Fig. S19**). However, there are no qualitative differences in the size or aspect ratio of the goethite needles (**Fig. S19**), which suggest that the small loss of surface area is primarily due to loss of porosity and surface roughness. Selected area electron diffraction patterns of annealed goethite needles and fast Fourier transforms of unannealed goethite needles display satellite peaks, indicating periodic and ordered distribution of stacking disorder (**Fig. S19**). However, these peaks become more smeared out into streaks with longer annealing (**Fig. S19**), suggesting that the stacking disorder is gradually lessening.

Macroscopic binding experiments in 2 mM total chloride were repeated in order to compare the effect of defects on the adsorption behavior of Pd and Pt. If Pd and Pt adsorption behavior on goethite is driven by Fe vacancies, there should be less adsorption to annealed goethite on a surface-area normalized basis. While there is less adsorption of both Pd and Pt on a per-mass basis (**Fig. S20**), this reflects the lower surface area in each experiment (**Table S1**). When adsorption is normalized to the available surface area in each experiment, there is no difference in adsorption between annealed vs. unannealed goethite (**Fig. S20**). Longer annealing time also does not affect Pd adsorption to goethite (**Fig. S20**). These findings suggest that the defects targeted by the annealing procedure (primarily near-surface Fe site vacancies) play no role, or are not the limiting factor, in Pt or Pd adsorption nor their high affinity for goethite.

Figures

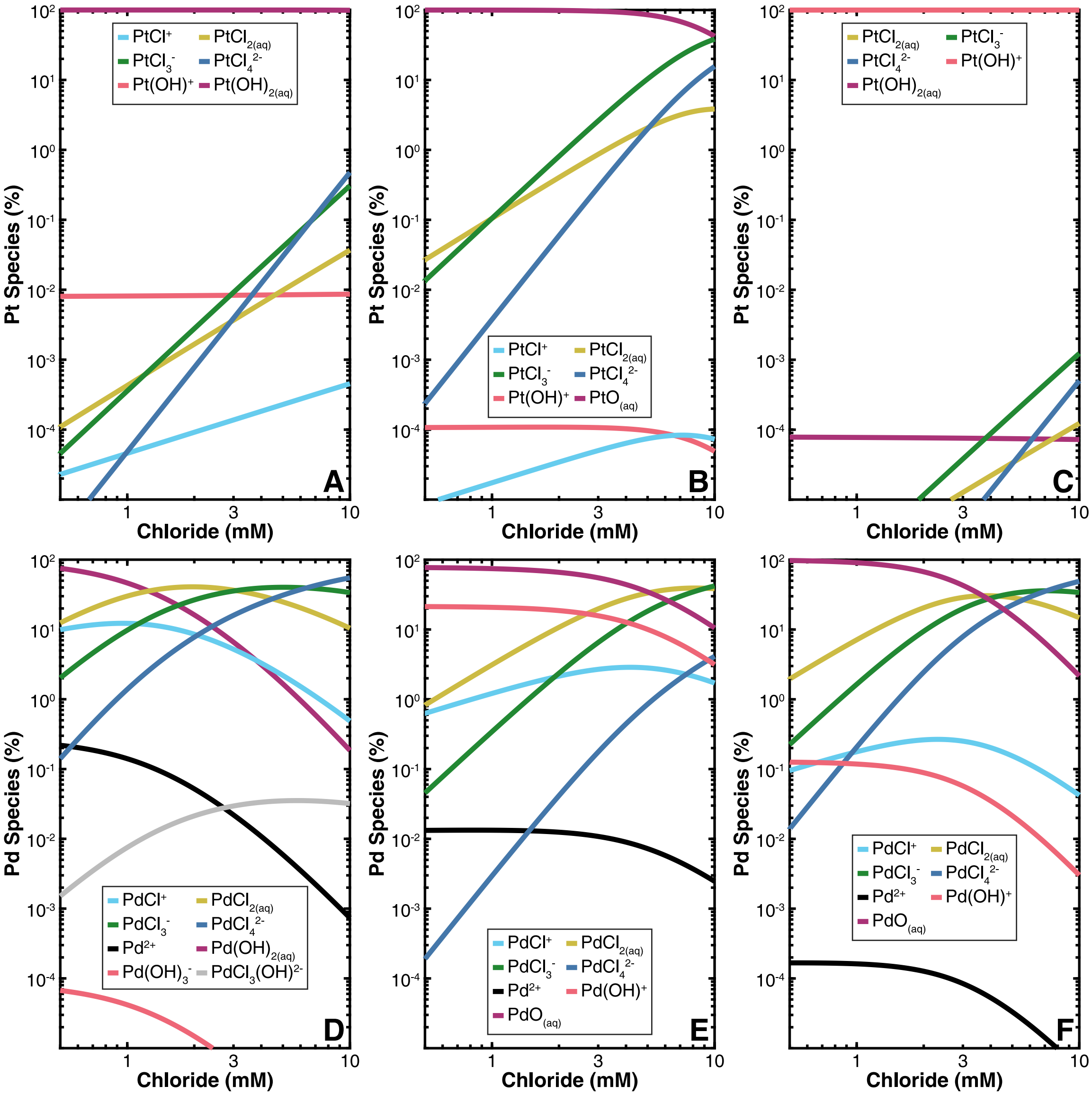

Fig. S1. The relative concentrations of (**A-C**) Pt(**II**) and (**D-F**) Pd(**II**) species at pH 4 as a function of increasing chloride from 0.5 to 10 mM predicted using equilibrium constants from (**A**) Mountain and Wood (26) and Wood et al. (27); (**B**) Sassani and Shock (28); (**C**) Azaroual et al. (23); (**D**) Rai et al. (29); (**E**) Tagirov et al. (31); and (**F**) Sassani and Shock (30). Note that $PdO_{(aq)}$ and $PtO_{(aq)}$ are equivalent to $Pd(OH)_{2(aq)}$ and $Pt(OH)_{2(aq)}$, respectively. Species that represent less than 0.00001% of the overall Pt speciation are not shown. Note that the speciation model based on the equilibrium constants provided by Rai et al. (29) used the recommended lower bound of log K = 5.42 (for the dissociation reaction).

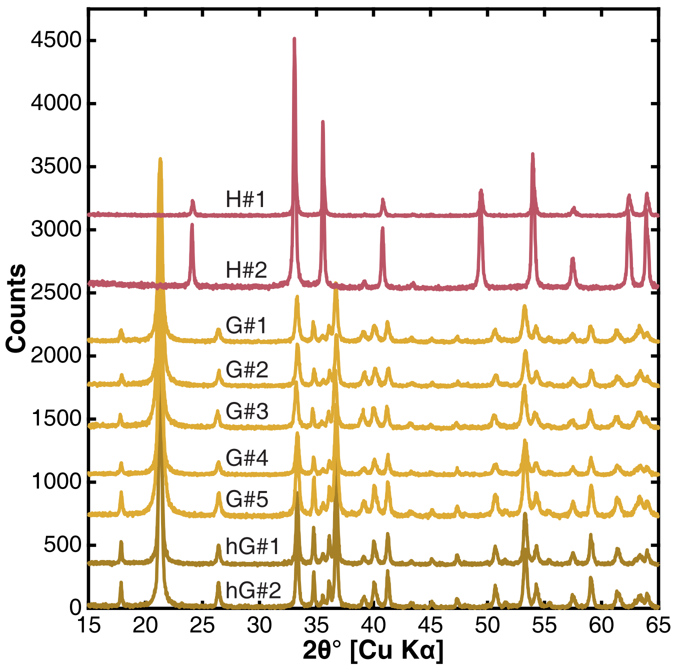

Fig. S2. X-ray diffraction patterns of synthesized goethite (G), hydrothermally-annealed goethite (hG), and hematite (H) batches used in this study (**Table S1**). Differences in intensity are due to different XRD pattern collection parameters.

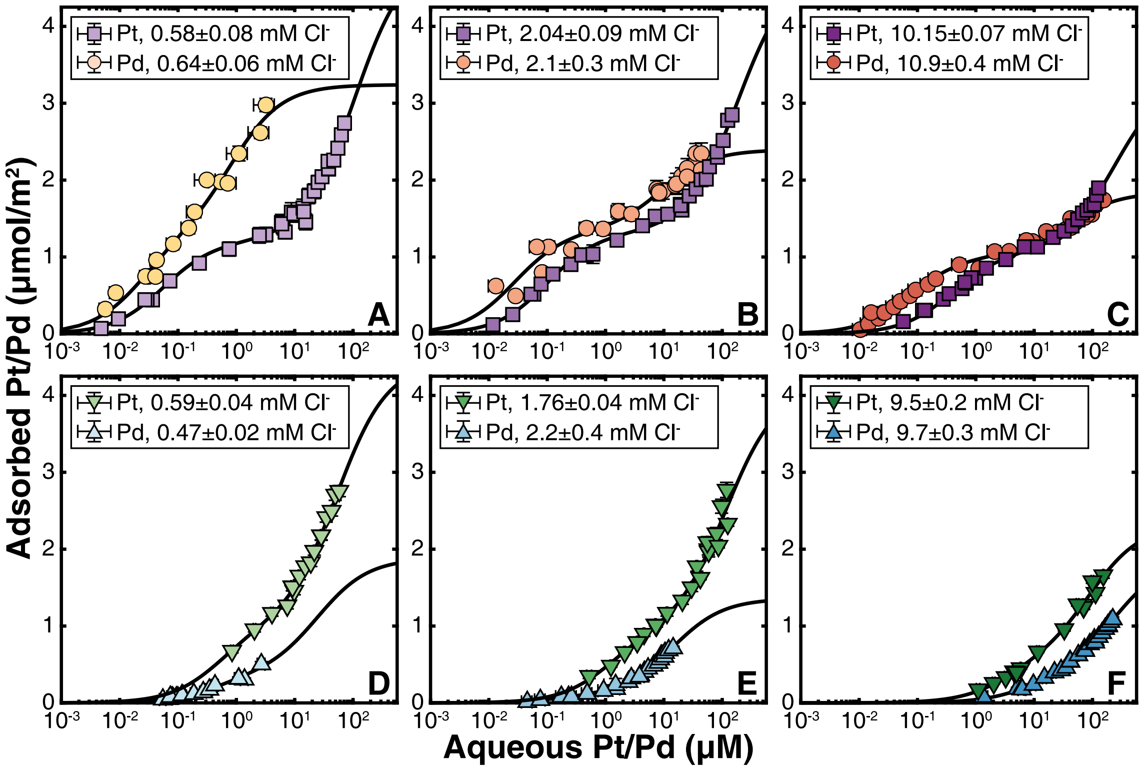

Fig. S3. Palladium(**II**) and platinum(**II**) surface coverage on (**A-C**) goethite and (**D-F**) hematite at pH 4±0.1 compared to the final dissolved Pd or Pt in approximately (**A,C**) 0.5, (**B,E**) 2, and (**C,F**) 10 mM total chloride. Corresponding Langmuir isotherm fits (**Table S2**) are shown as solid lines. Experimental errors smaller than symbols are not shown. A subset of each Pt-goethite dataset has been corrected; see the Supplementary Information, **Fig. S22**, and **Table S2** for more details. Data on Pd adsorption to hematite is from prior work (2) but newly refit with two-component Langmuir isotherms.

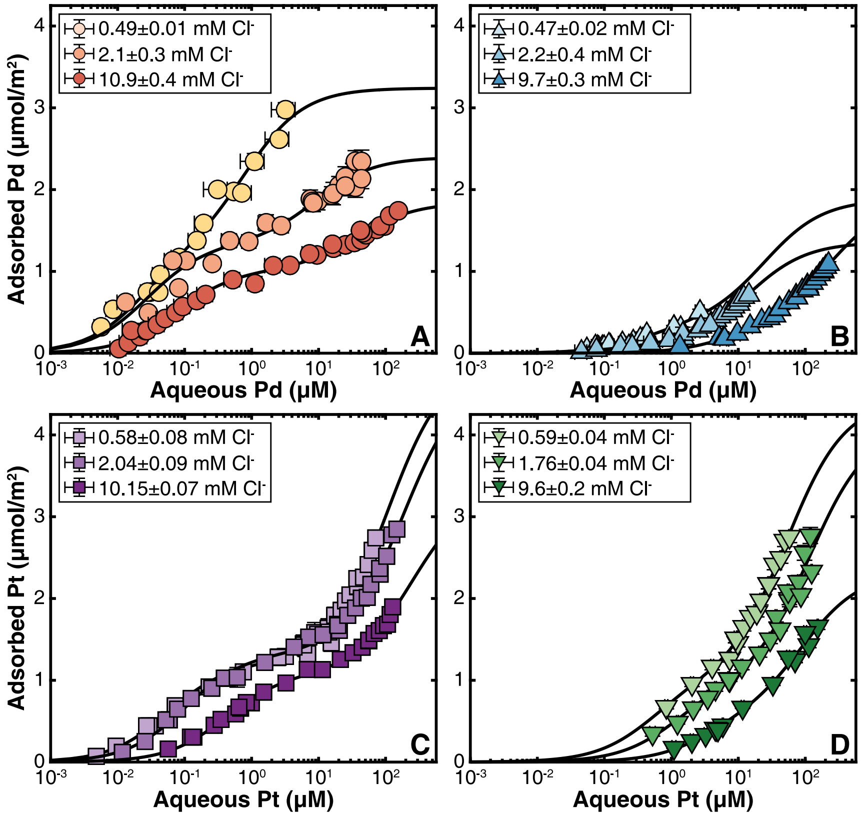

Fig. S4. (**A,B**) Palladium and (**C,D**) platinum adsorption to (**A,C**) goethite or (**B,D**) hematite compared to final measured dissolved Pd or Pt at pH 4±0.1 in the presence of varying amounts of chloride. Corresponding Langmuir isotherm fits (**Table S2**) are shown as solid lines. Experimental errors smaller than the symbols are not shown. A subset of each Pt-goethite dataset has been corrected; see the Supplementary Information, **Fig. S22**, and **Table S2** for more details. Data on Pd adsorption to hematite (**B**) is from previously published work (2), but newly fit with dual Langmuir isotherms (**Table S2**).

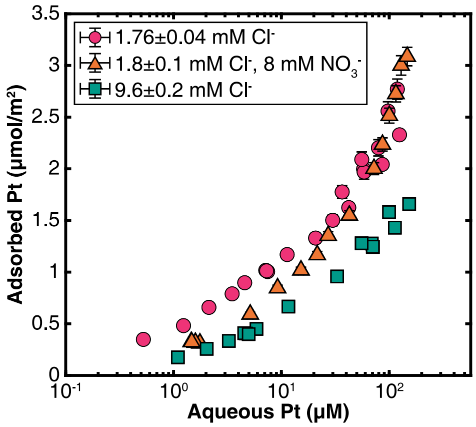

Fig. S5. Platinum(II) adsorption to hematite at pH 4±0.1 compared to the final measured dissolved Pt, evaluating the effect of changing ionic strength (adding $NaNO_3$) compared to changing the amount of dissolved chloride. Errors smaller than the symbols are not shown.

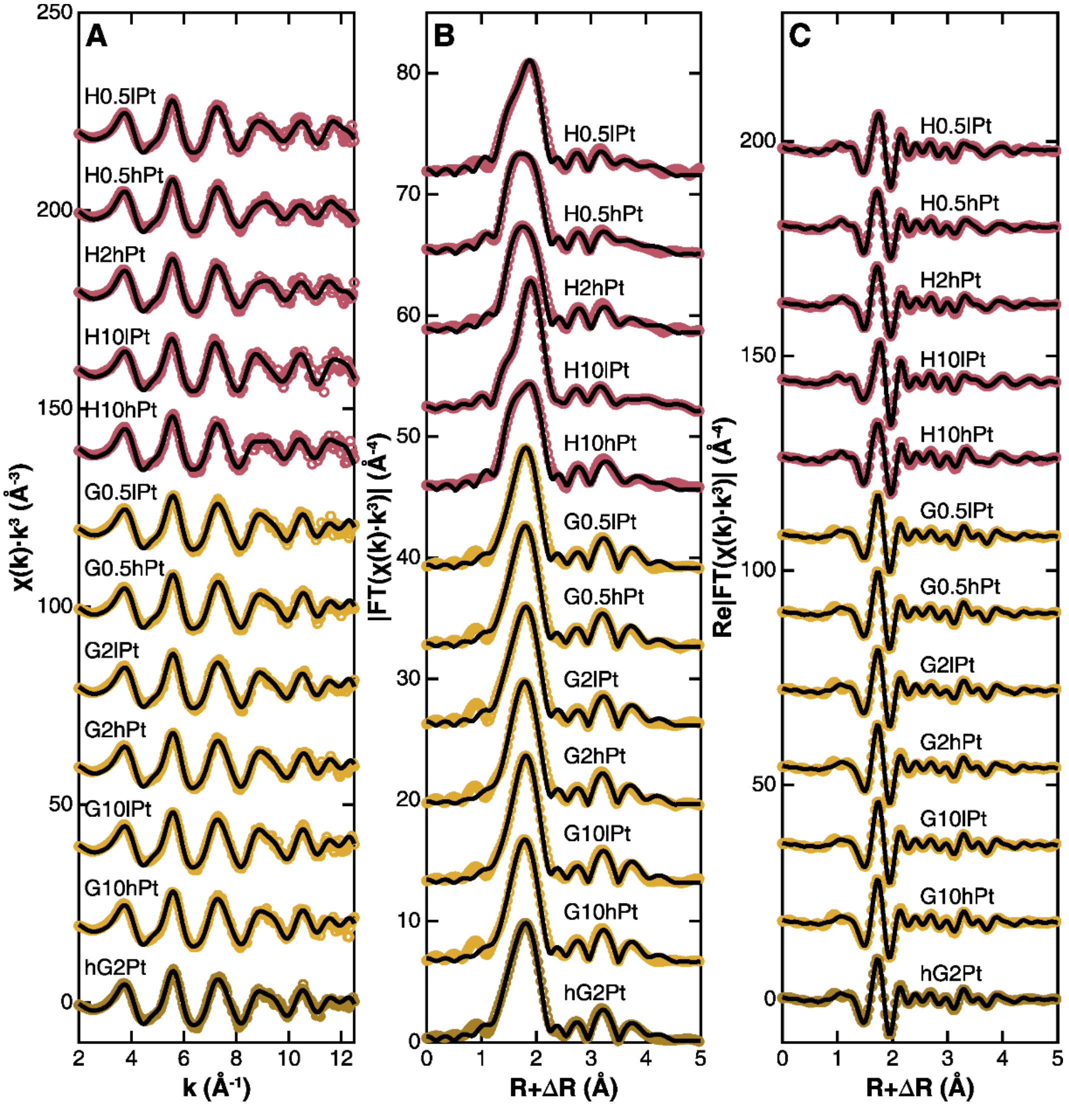

Fig. S6. Data (symbols) and structural model fit (line) to the (**A**) platinum L_3-edge spectra, (**B**) Fourier transform magnitudes, and (**C**) real components of the Fourier transforms for samples of Pt adsorbed to hematite, goethite, and hydrothermally-annealed goethite. Detailed sample information is provided in **Table S4**.

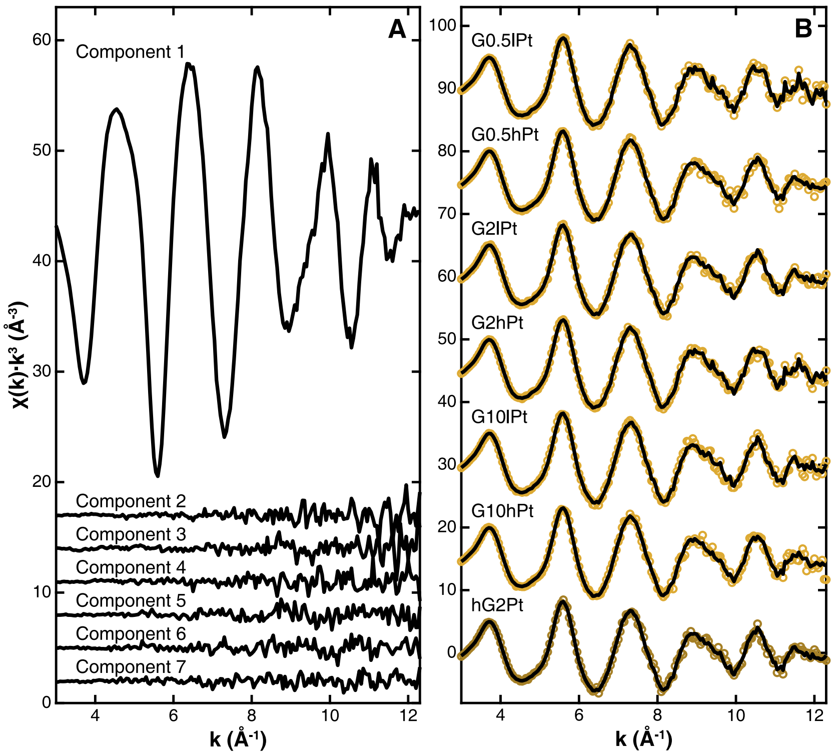

Fig. S7. Results of the principal component analysis of the EXAFS spectra of platinum adsorbed to goethite and hydrothermally-annealed goethite. (**A**) Principal components derived from analysis. (**B**) Reconstruction (line) of the experimental spectra (symbols) using two principal components. Detailed sample information is provided in **Table S4**.

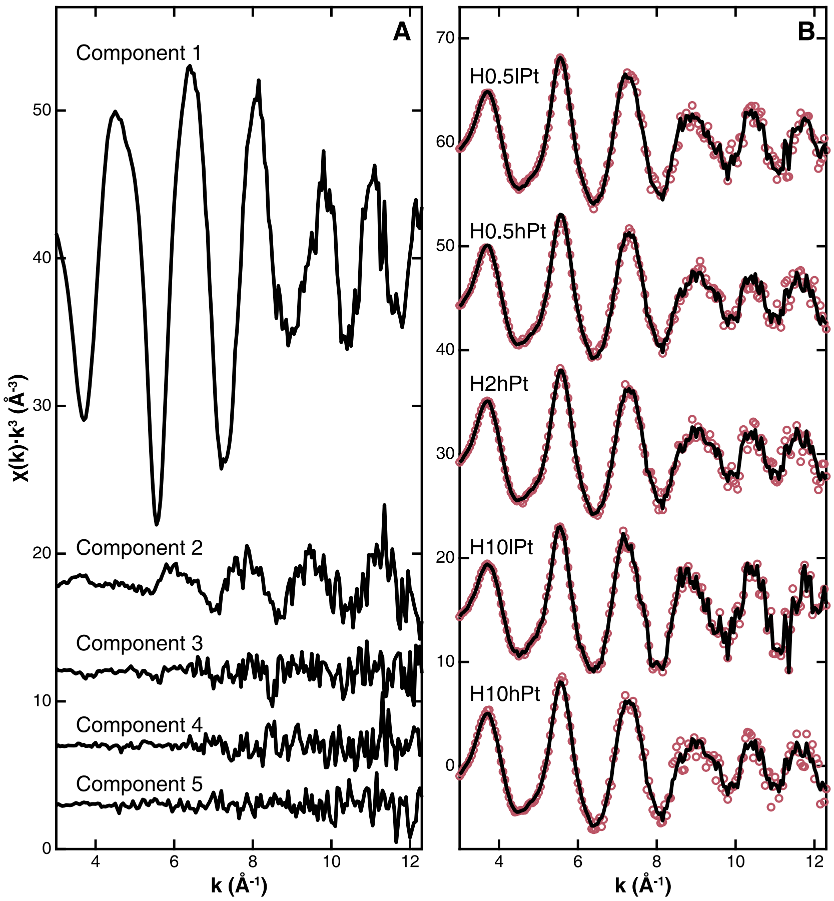

Fig. S8. Results of the principal component analysis of the EXAFS spectra of platinum adsorbed to hematite. (**A**) Principal components derived from analysis. (**B**) Reconstruction (line) of the experimental spectra (symbols) using two principal components. Detailed sample information is provided in **Table S4**.

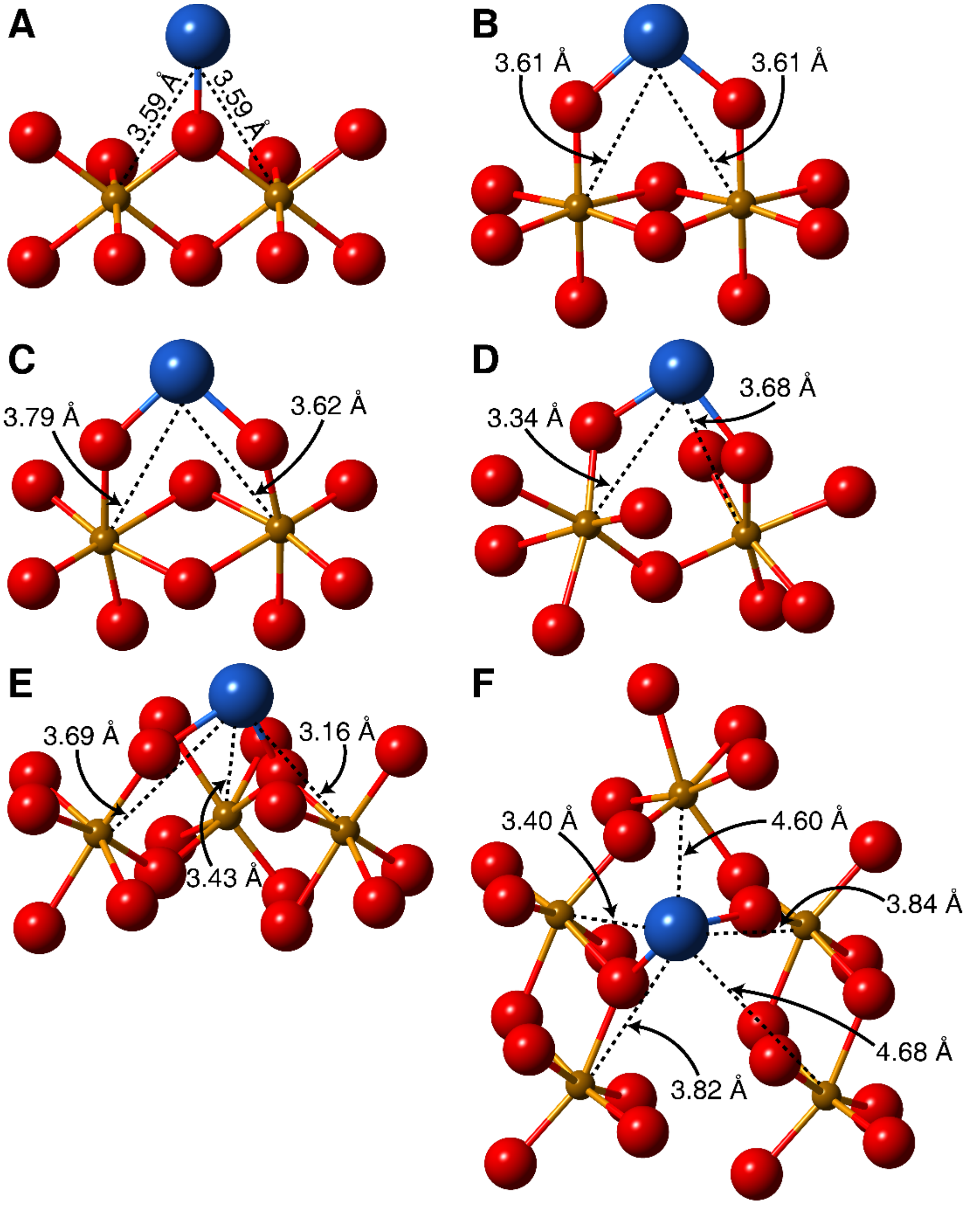

Fig. S9. Possible binding geometries for Pd/Pt surface complexes consistent with the EXAFS results and the coordination of octahedra in the goethite surface: (**A**) monodentate complex bound to a doubly-coordinated oxygen site on the (101) surface; (**B**) bidentate complex bridging between the corners of two edge-sharing iron octahedra on the (101) surface; (**C**) bidentate complex bridging between the corners of two edge-sharing iron octahedra on the (210) surface; (**D**) bidentate complex bridging between the corners of two corner-sharing iron octahedra on the (210) surface; (**E**) bidentate complex formed over with a cluster of three iron octahedra on the (210) surface; (**F**) bidentate complex formed over with a cluster of five iron octahedra on the (210) surface. All Pd/Pt-O bond lengths are 2.00-2.02 Å. Note that the interatomic distances shown are approximate and variations of ±0.1 Å are possible with slight changes in bond lengths and angles. Additional relaxations at the surface may further alter these distances.

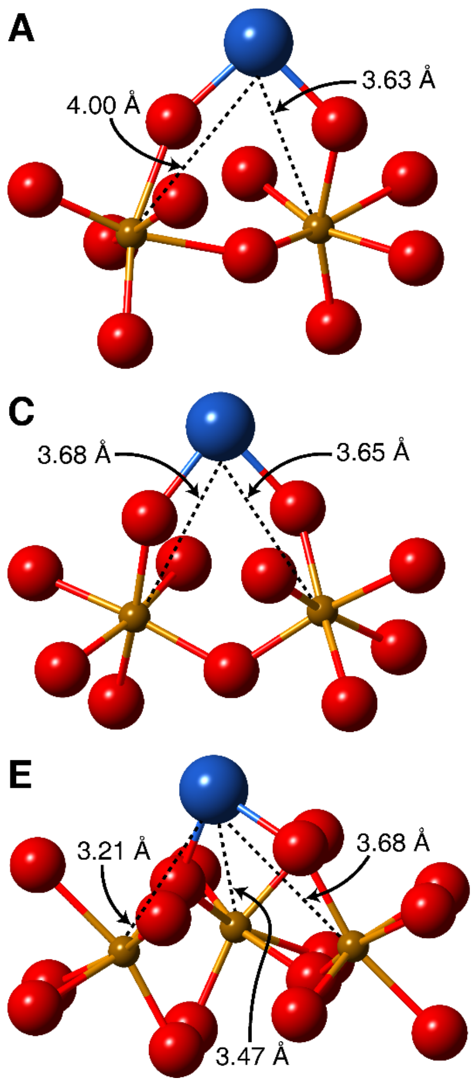

Fig. S10. Possible binding geometries for Pt surface complexes consistent with the EXAFS results and the coordination of octahedra in the hematite surface: (**A**) bidentate complex bridging between the corners of two corner-sharing iron octahedra on the full termination of the (012) surface; (**B**) bidentate complex bridging between the corners of two corner-sharing iron octahedra on the half termination of the (012) surface; (**C**) bidentate complex bridging between the corners of two corner-sharing iron octahedra on the (110) surface; (**D**,**E**) bidentate complexes formed over with a cluster of three iron octahedra on the (110) surface. All Pt-O bond lengths are 2.00-2.02 Å. Note that the interatomic distances shown are approximate and variations of ±0.1 Å are possible with slight changes in bond length and angle. Additional relaxations at the surface may further alter these distances.

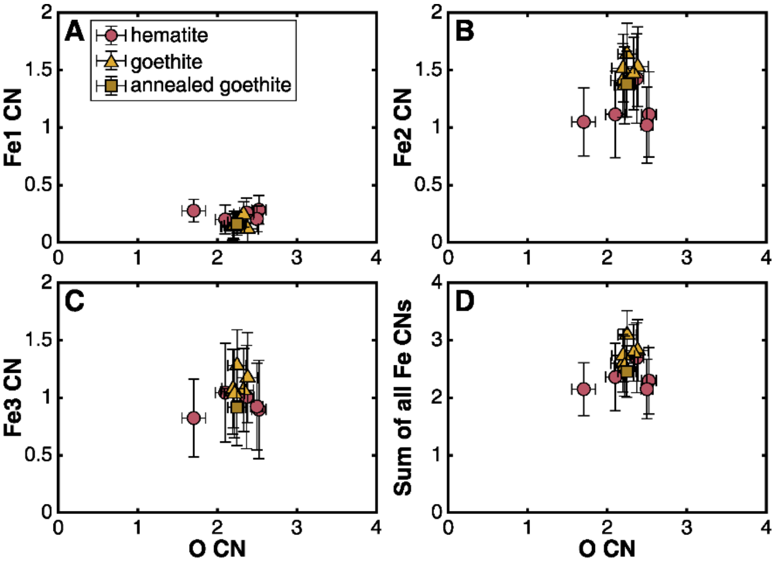

Fig. S11. Trends in structural model fits to all Pt EXAFS spectra (see **Tables S7**,**S8**) comparing the O coordination number (CN) to the number of (**A**) edge-sharing Fe neighbors at 3.0-3.1 Å (Fe1); (**B**) corner-sharing Fe neighbors at 3.7 Å (Fe2); (**C**) corner-sharing Fe neighbors at 3.9 Å (Fe3); and (**D**) the total number of Fe neighbors.

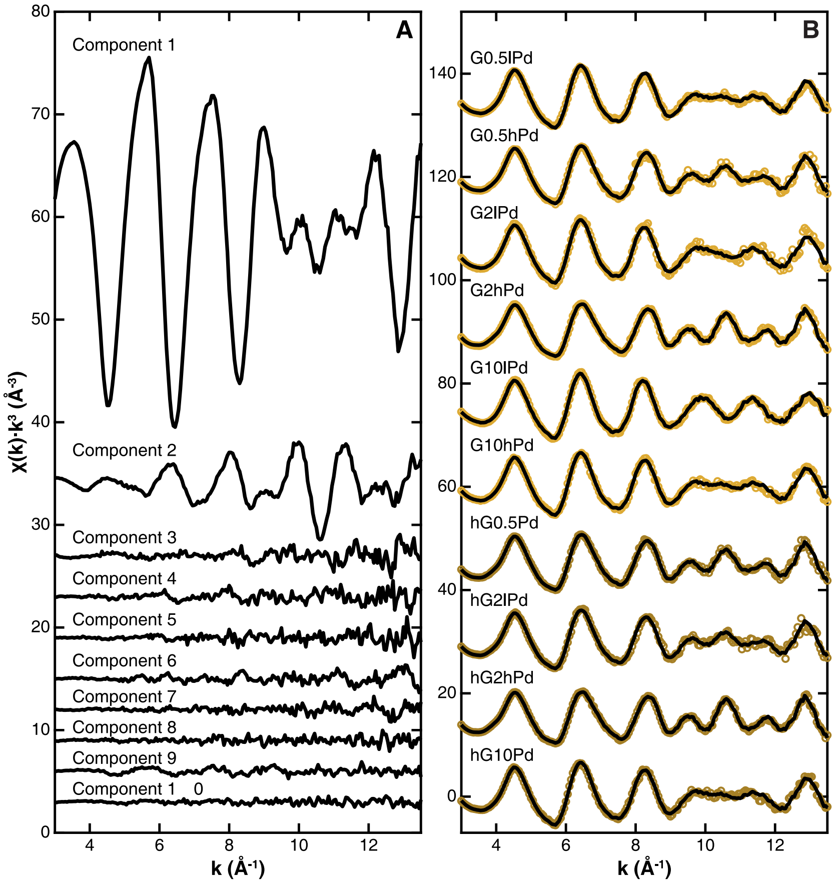

Fig. S12. Results of the principal component analysis of the EXAFS spectra of Pd(**II**) adsorbed to goethite and hydrothermally-annealed goethite. (**A**) Principal components derived from analysis. (**B**) Reconstruction (line) of the experimental spectra (points) using two principal components. Detailed sample information is provided in **Table S4**.

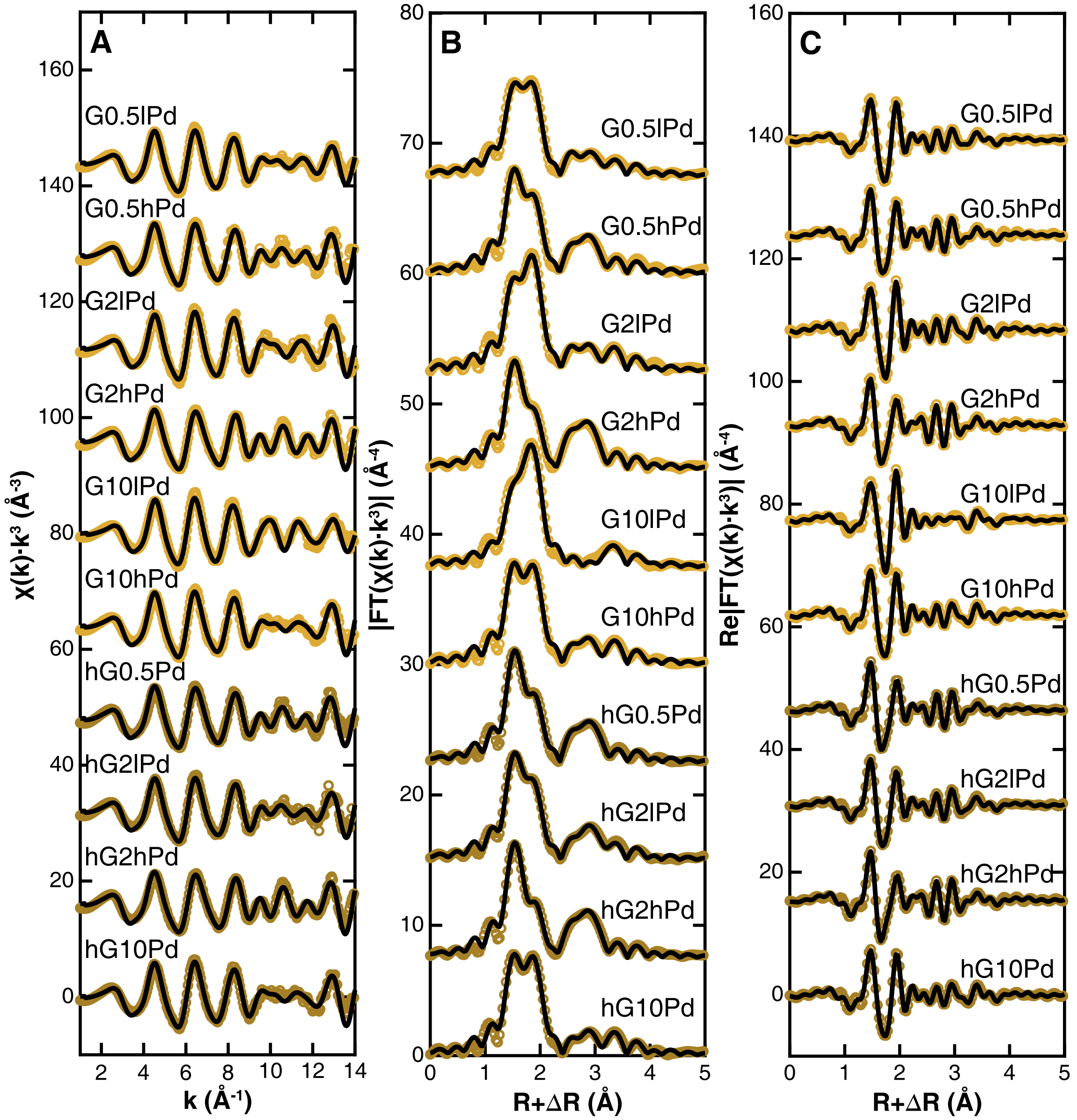

Fig. S13. Data (symbols) and structural model fit (line) to the Pd K-edge spectra (**A**), Fourier transform magnitudes (**B**), and real components of the Fourier transforms (**C**) of samples of Pd(**II**) adsorbed to goethite and hydrothermally-annealed goethite. Detailed sample information is provided in **Table S4**.

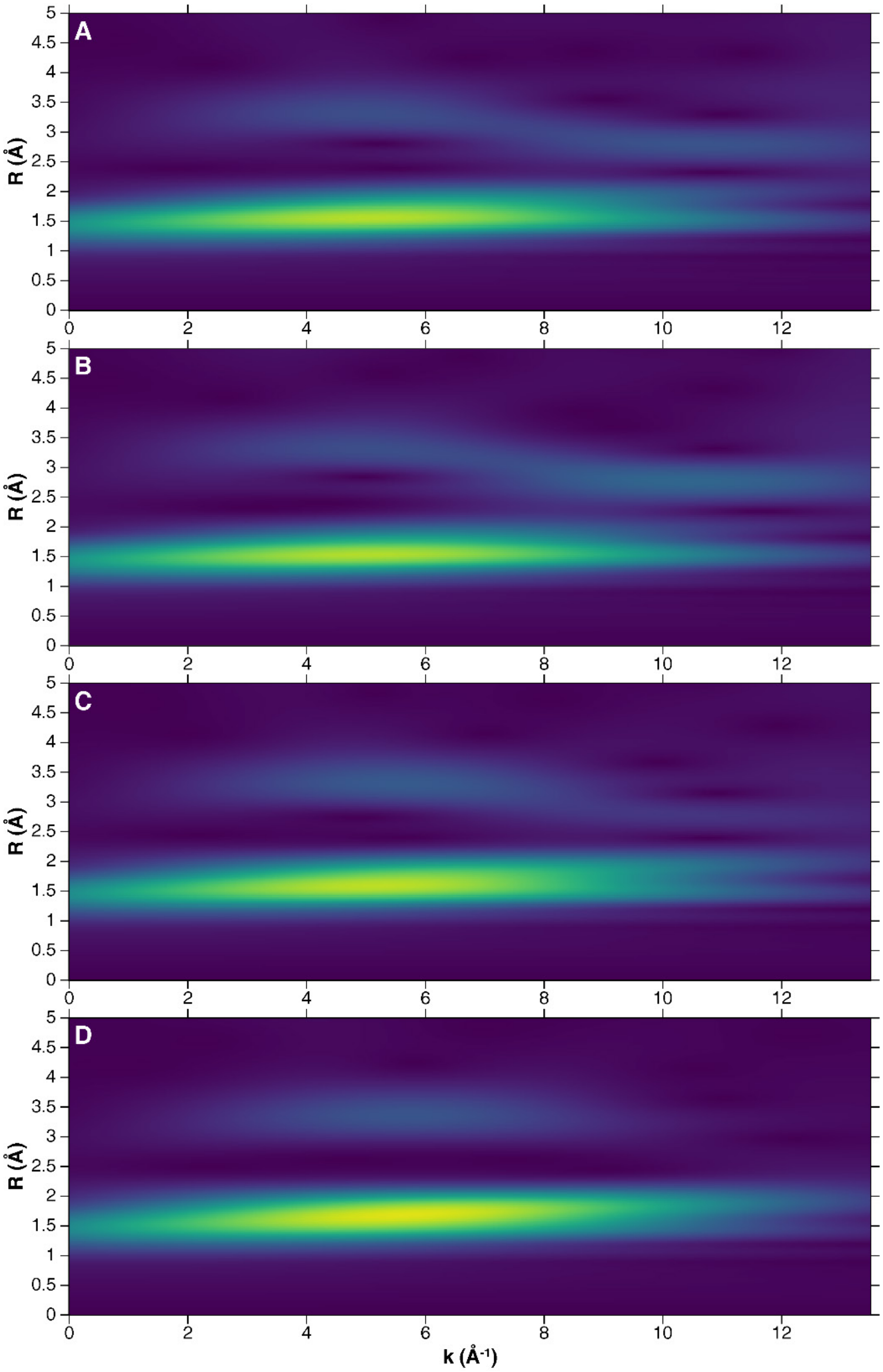

Fig. S14. Wavelet transforms of (**A**) G0.5hPd, (**B**) G2hPd, (**C**) G10hPd, and (**D**) G10lPd. Note the feature between 2.5 and 3 Å (R+ΔR) and 9 and 13.5 $Å^{-1}$ (*k*) observed in all spectra except for G10lPd.

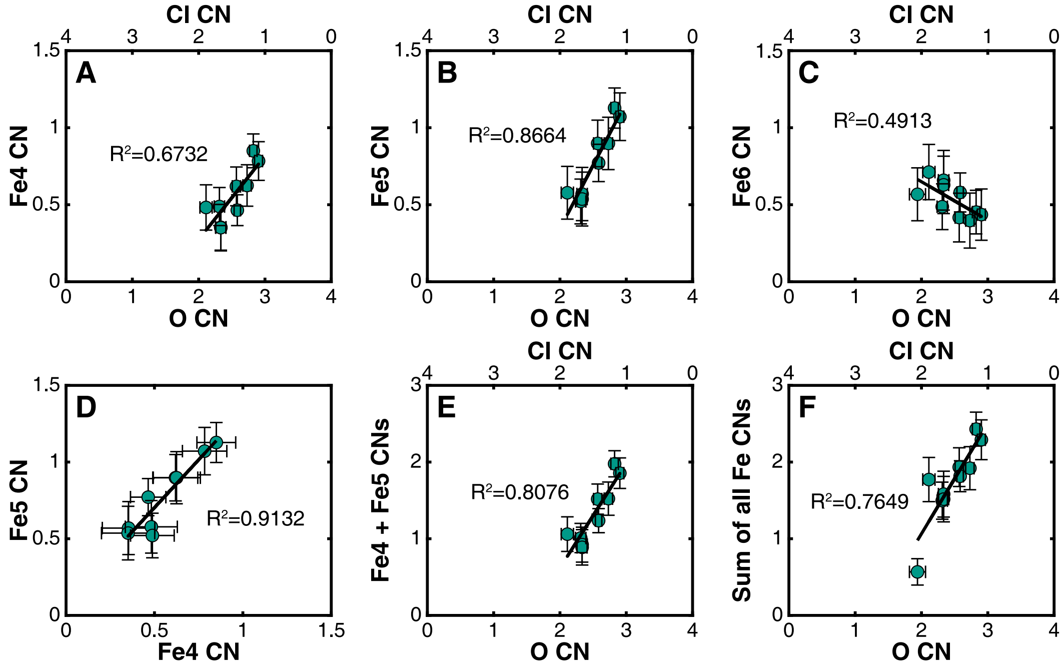

Fig. S15. Correlations between the O coordination number (CN) and the CNs fit to Pd EXAFS spectra of Pd adsorbed to goethite and hydrothermally-annealed goethite for (**A**) Fe neighbors at 2.9 Å (Fe4), resulting from Pd binding to the edges of iron octahedra; (**B**) Fe neighbors at 3.2 Å (Fe5), resulting from Pd binding to the edges of iron octahedra; (**C**) Fe neighbors at 3.6 Å (Fe6), resulting from Pd binding to the corners of iron octahedra; (**E**) all Fe neighbors at < 3.2 Å (Fe4 and Fe5), resulting from Pd binding to the edges of iron octahedra; and (**F**) all Fe neighbors. (**D**) The correlation between CNs for Fe neighbors at edge-sharing distances is shown. Unweighted linear regression lines and associated R^2 values are shown for all correlations. Full fitting results are reported in **Table S10**. Note that sample G10lPd is only included in (**A**) and (**F**); Fe neighbors at edge-sharing distances were not fit to that spectrum.

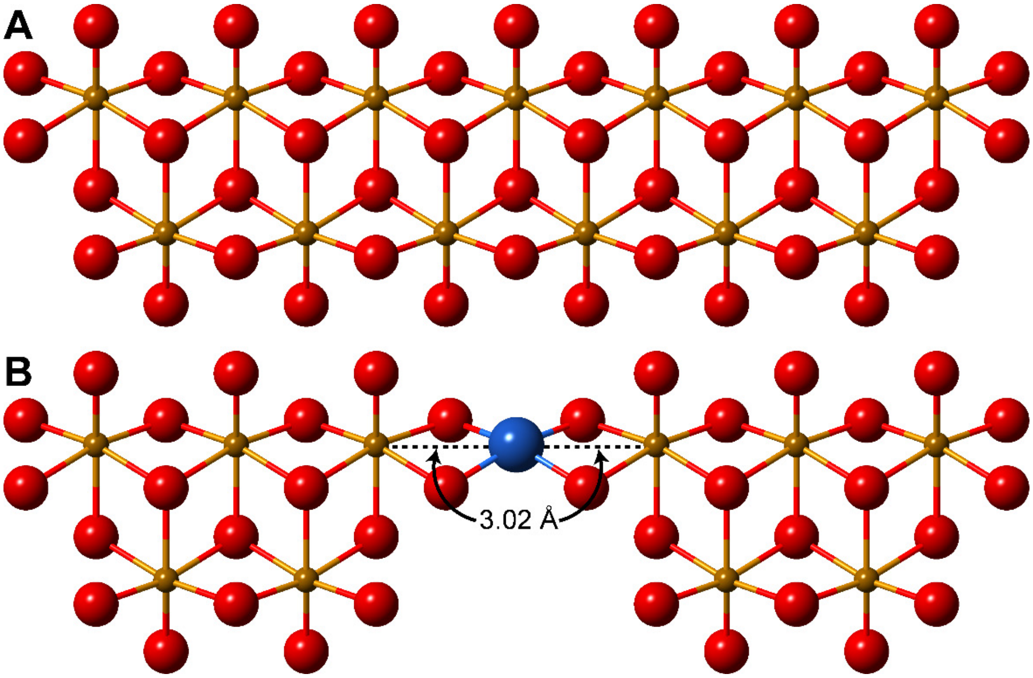

Fig. S16. A view of a goethite structural chain (**A**) and with a Pd atom replacing an Fe site and additional Fe vacancies (**B**). The Pd position is shifted slightly from the Fe position and two short Fe-O bonds were lengthened to give four consistent Pd-O bond lengths.

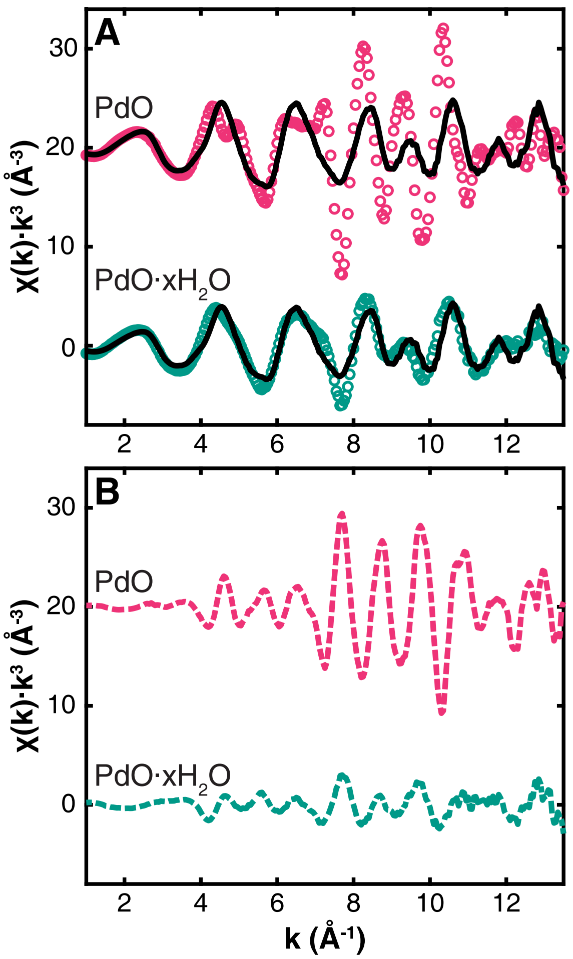

Fig. S17. (**A**) Data (symbols) and target transformations (line) using 2 components from the PCA of all Pd-goethite spectra and (**B**) the corresponding resulting residuals for PdO (2) and hydrous PdO (78).

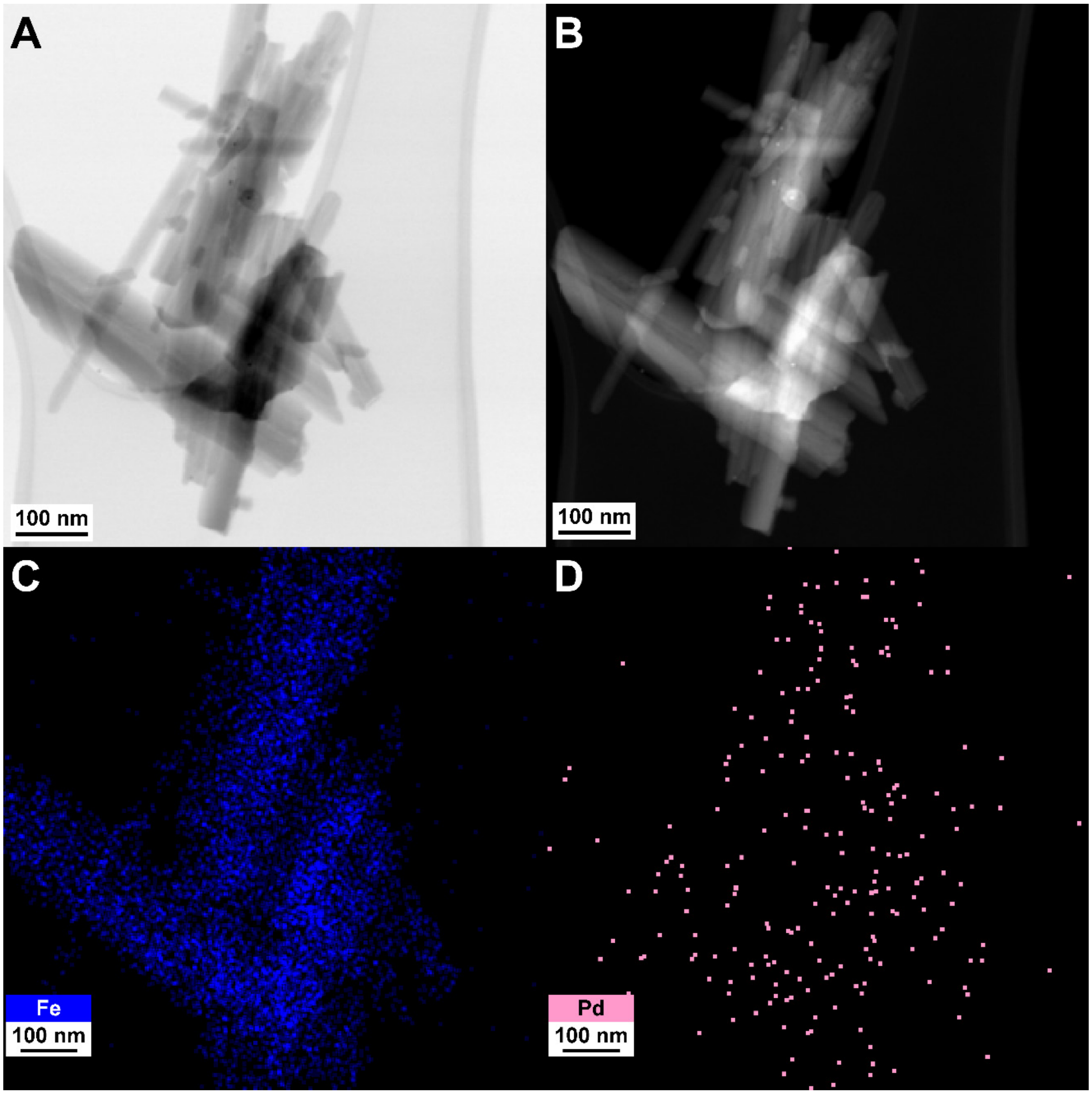

Fig. S18. Scanning transmission electron microscopy of Pd reacted with goethite in (**A**) bright-field and (**B**) dark-field and corresponding (**C**) Fe and (**D**) Pd element maps.

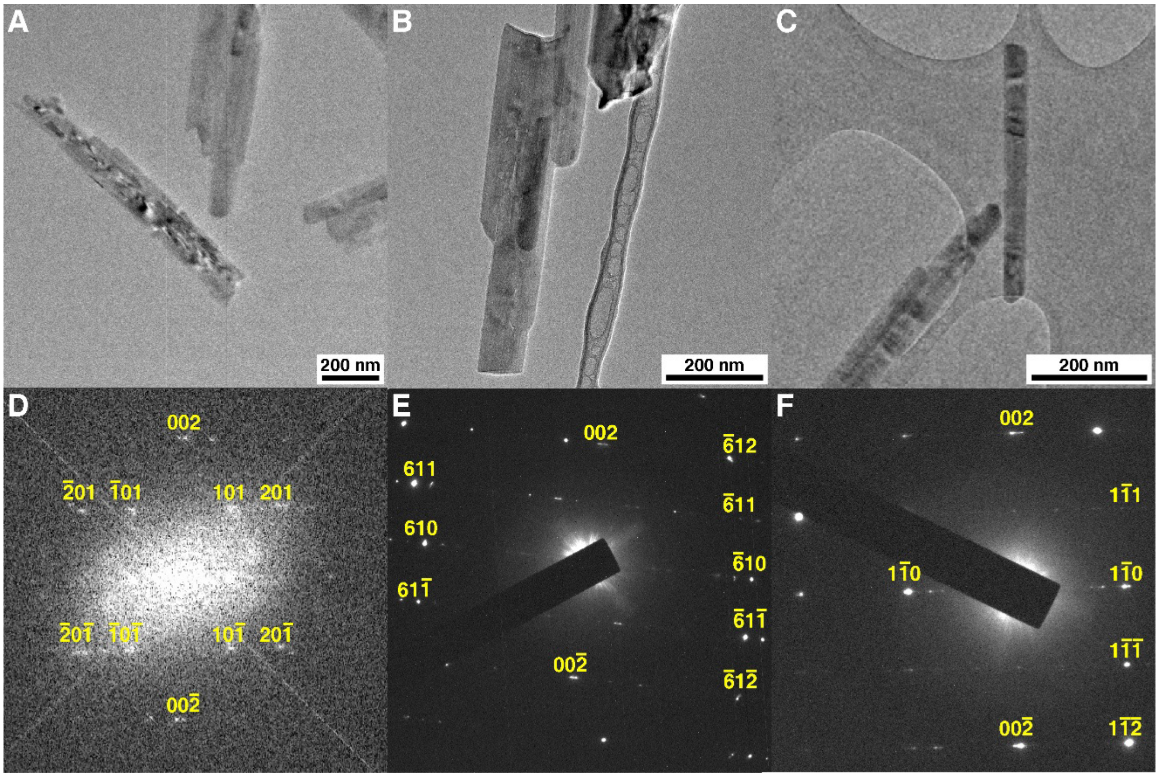

Fig. S19. Transmission electron microscopy of (**A**) unannealed goethite; (**B**) goethite annealed for 44 hours (hG#1); and (**C**) goethite annealed for 1 week (hG#2). Representative fast Fourier transform (**D**) or selected area electron diffraction (**E,F**) for each goethite sample are shown. Satellite peaks, indicating periodic and ordered distribution of stacking disorder become smeared out with longer annealing time, indicating the gradual disappearance of stacking disorder.

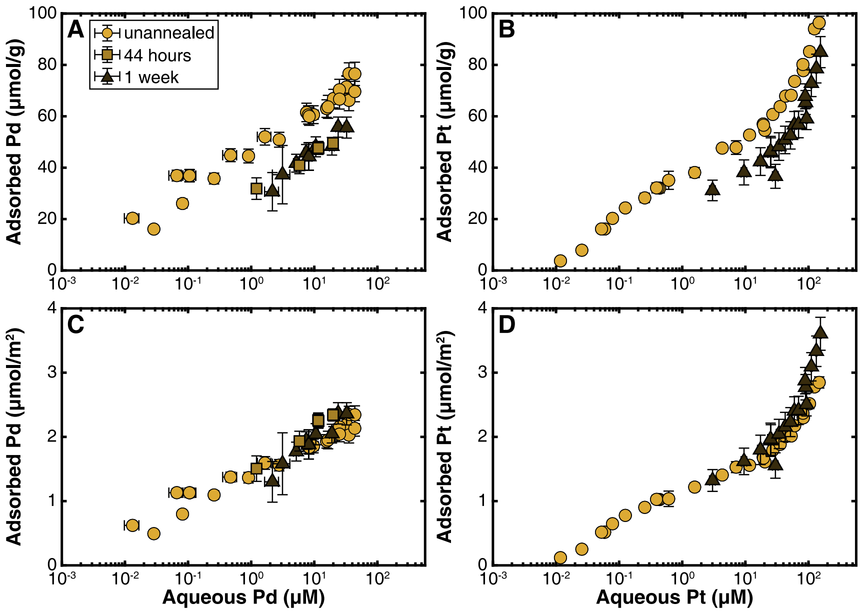

Fig. S20. (**A,C**) Palladium(**II**) and (**B,D**) Pt(**II**) adsorption to goethite and hydrothermally-annealed goethite in approximately 2 mM total chloride at pH 4±0.1. Adsorption is shown on a (**A,B**) per-mass basis and (**C,D**) normalized to surface area. Errors smaller than the symbols are not shown. Note that some of the Pt(**II**) adsorption data has been scaled when plotted as a function of surface area; see the Supplementary Information, **Fig. S22**, and **Table S2** for more details.

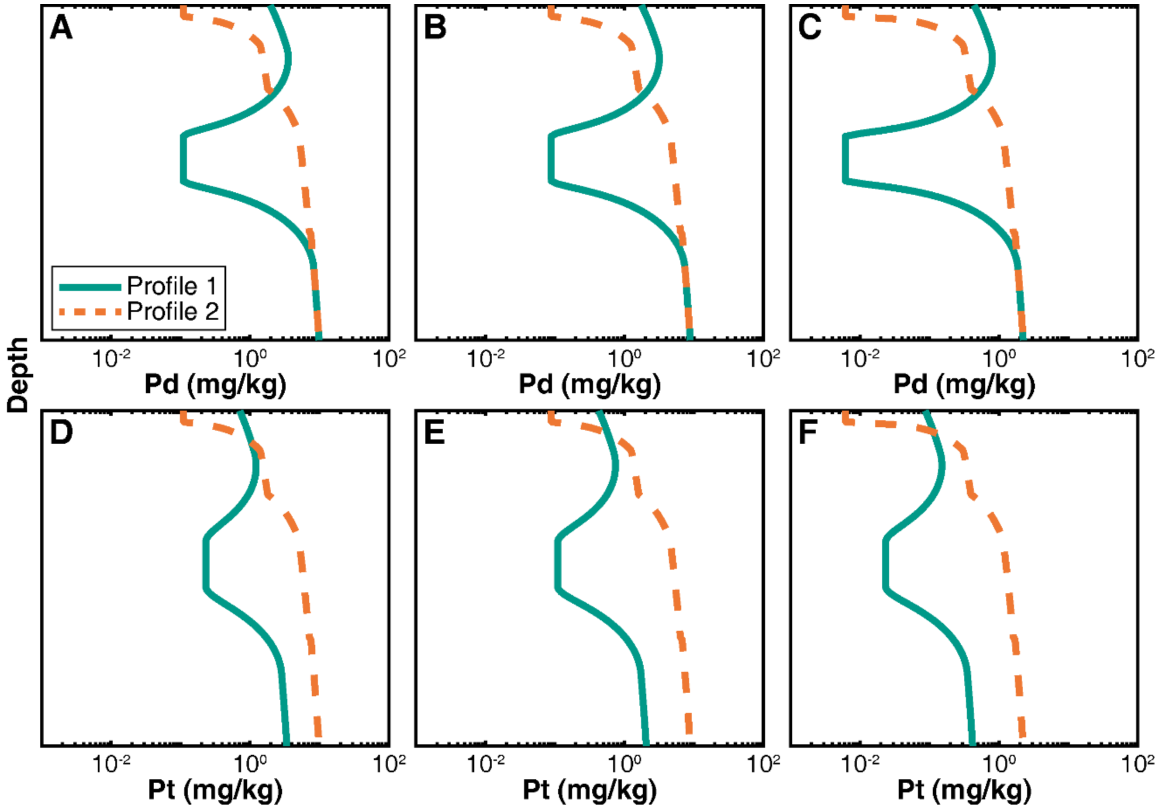

Fig. S21. Calculated (**A-C**) Pd (**D-F**) Pt concentrations (in ng/g) with depth for two different iron (oxyhydr)oxide profiles in approximately (**A,D**) 0.5, (**B,E**) 2, or (**C,F**) 10 mM total chloride. See **Fig. 3** for more details on the two profiles.

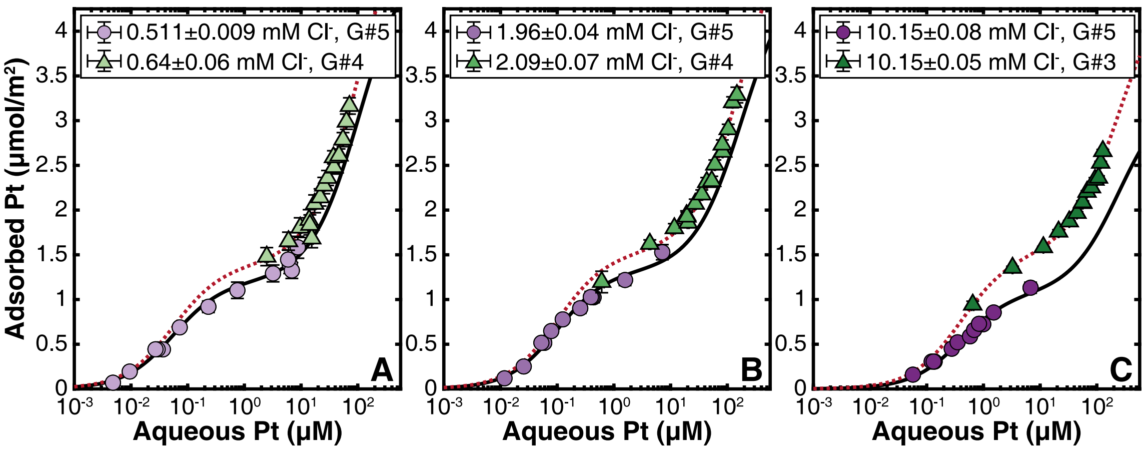

Fig. S22. Platinum(**II**) adsorption to goethite at pH 4±0.1 compared to the final measured dissolved Pt in approximately (**A**) 0.5, (**B**) 2, and (**C**) 10 mM total chloride. Two separate experiments, collected with different batches of goethite, are shown. Errors smaller than symbols are not shown. Corresponding Langmuir isotherm fits are shown as lines. The dual Langmuir isotherm was fit to both datasets simultaneously with an additional scaling value fit to describe the difference between the datasets.

Tables

Table S1. Mineral characterization details for mineral batches used in study.

Batch Name	Mineral[a]	Annealing Time[b]	Measured Surface Area (m^2/g)[c]	Corresponding Experiments
G#1	goethite	N/A	32.6	Pd isotherms with 2 and 10 mM Cl
G#2	goethite	N/A	23.5	Pd isotherm with 0.5 mM Cl; EXAFS samples G0.5lPd, G2lPd, G2hPd, G10lPd
G#3	goethite	N/A	28.9	Pt isotherm with 10 mM Cl; EXAFS samples G0.5hPd, G10hPd
G#4	goethite	N/A	29.3	Pt isotherms with 0.5 and 2 mM Cl; EXAFS sample G2lPt
G#5	goethite	N/A	31.3	EXAFS samples G0.5lPt, G0.5hPt, G2hPt, G10lPt, G10hPt; additional low concentration samples for Pt isotherms with 0.5, 2, and 10 mM Cl
hG#1	goethite	44 hours	21.2	Pd isotherm with 2 mM Cl; EXAFS samples hG0.5Pd, hG2hPd, hG10Pd
hG#2	goethite	1 week	23.6	Pd and Pt isotherm with 2 mM Cl; EXAFS samples PdhG2l, PthG2
H#1	hematite	N/A	23.9	Pt isotherms with 0.5, 2, and 10 mM Cl; Pt isotherm with 2 mM Cl and 8 mM NO_3
H#2	hematite	N/A	N/A	EXAFS samples H0.5lPt, H0.5hPt, H2hPt, H10lPt, H10hPt

[a]Mineralogy verified with XRD (see **Fig. S2**).
[b]Hydrothermally-annealed goethite batches were aged in ultrapure water at 150°C for the specified amount of time.
[c]BET specific surface area. Surface area was not measured for mineral batches that were solely used to prepare EXAFS samples.

Table S2. Langmuir parameters from isotherms fit to the uptake for the Pd(II) and Pt(II) adsorption experiments at pH 4±0.1.

Element/Mineral	[Cl] (mM)[a]	K_1 (μM^{-1})[b]	Γ_{max1} ($\mu mol/m^2$)[c]	K_2 (μM)[b]	Γ_{max2} ($\mu mol/m^2$)[c]	Scaling Factor[d]
Pd/Goethite	0.49±0.01	43(9)[e]	1.3	1.2(4)	1.9(3)	-
	2.1±0.3	40(10)	1.3(1)	0.11(5)	1.1(1)	-
	10.9±0.4	13(1)	1.01(4)	0.028(8)	0.81(7)	-
Pd/Hematite[f]	0.47±0.02	2.1(2)	0.38	0.040(7)	1.5	-
	2.2±0.4	6(5)	0.10(3)	0.07(2)	1.3(1)	-
	9.7±0.3	0.11(3)	0.38(8)	0.004(1)	1.5(2)	-
Pt/Goethite	0.58±0.08	18(3)	1.21(4)	0.009(4)	3.7(8)	1.15(4)
	2.04±0.09	10(1)	1.32(4)	0.005(2)	3.4(5)	1.15(4)
	10.15±0.07	2.5(2)	1.07(4)	0.004(2)	2.3(6)	1.40(4)
Pt/Hematite	0.59±0.04	1.9(8)	1.0(1)	0.019(6)	3.4(4)	-
	1.76±0.04	0.9(6)	1.0(2)	0.009(6)	3.2(8)	-
	9.5±0.5	0.3(2)	0.6(3)	0.011(8)	1.7(3)	-

[a]Chloride is reported as the average final aqueous concentration in experimental samples, plus or minus one standard deviation.
[b]K is an affinity constant that represents the favorability of adsorption.
[c]Γ_{max} is the maximum surface area-normalized binding capacity.
[d]For isotherms that were constructed using two experiments that used different batches of goethite, both datasets were fit simultaneously but a multiplicative factor was applied to scale the isotherms between batches. By convention, the scaling factor was applied to the higher Pt coverage dataset.
[e]Estimated standard deviations as errors in the last digit are given in parentheses. Parameters that were constrained during fitting are listed with no uncertainties.
[f]Data on Pd adsorbed to hematite is from prior work (2), newly refit with dual Langmuir isotherms.

Table S3. Measured zeta potential for goethite and hematite at pH 4±0.1 in the presence of varying concentrations of dissolved NaCl.

NaCl (mM)	Zeta Potential (mV)[a]	
	Goethite	Hematite[b]
0.5	27.0±0.4	45.3±0.4
2	23±2	45.1±0.4
10	25±1	49±2[c]

[a]Zeta potential is reported as the average and standard deviation of multiple measurements.
[b]Data for hematite previously reported (2).
[c]The zeta potential for hematite in 10 mM Cl^- is reported as the average, plus or minus the standard deviation, of six measurements.

Table S4. Details about EXAFS sample experimental conditions.

Sample[a]	Mineral Batch	Mineral Loading (g/L)	Sample Volume (mL)	Aqueous Chloride (mM)	Final Aqueous Pt or Pd (μM)	Adsorbed Pt or Pd (μmol/m²)[b]	Sample Prep Method[c]
H0.5lPt	H#2	5	100	0.51	3.39	1.1	[4]
H0.5hPt	H#2	4	100	0.57	17.2	1.8	{4]
H2hPt	H#2	4	100	1.97	64.9	2.2	[4]
H10lPt	H#2	8	50	9.50	22.5	0.86	[4]
H10hPt	H#2	4	100	9.34	136	1.6	[4]
G0.5lPt	G#5	1.5	100	0.51	0.60	1.1	[3]
G0.5hPt	G#5	1.5	100	0.54	18.3	1.7	[3]
G2lPt	G#4	4	50	1.93	2.14	1.3	[3]
G2hPt	G#5	3	50	1.98	32.7	1.8	[3]
G10lPt	G#5	3	50	10.0	10.9	1.1	[3]
G10hPt	G#5	3	50	10.0	65.9	1.5	[3]
hG2Pt	hG#2	4	50	2.14	21.2	1.6	[3]
G0.5lPd	G#2	0.8	100	0.49	0.03	0.80	[2]
G0.5hPd	G#3	0.8	50	0.48	0.06	1.0	[2]
G2lPd	G#2	4	50	1.75	0.17	1.2	[1]
G2hPd	G#2	2	100	1.88	12.9	1.9	[2]
G10lPd	G#2	4	50	11.0	0.12	0.62	[1]
G10hPd	G#3	1	50	10.2	45.0	1.5	[2]
hG0.5Pd	hG#1	0.8	50	0.50	0.11	1.3	[2]
hG2lPd	hG#2	2	100	1.82	1.43	1.4	[3]
hG2hPd	hG#1	1	50	1.98	17.3	2.0	[2]
hG10Pd	hG#1	1	50	10.3	54.1	1.5	[2]

[a]The element listed at the end of the sample name specifies whether Pd or Pt were used. Names that start with "H", "G", and "hG" are hematite, goethite, and hydrothermally-annealed goethite samples, respectively.

[b]Estimated by using the final aqueous Pt or Pd and calculating the adsorbed Pt or Pd using Langmuir isotherm values (see **Table S2**) for the hematite or non-annealed goethite isotherms at the approximate chloride concentrations.

[c]Sample preparation methods are described in more details in the Supplementary Information. [1]: sample centrifuged at high speeds and then scraped from container; [2] sample collected and packed on filter membrane; [3] sample collected on filter membrane and then packed in standard sample holder; [4] sample centrifuged at slower speeds and then scraped from container.

Table S5. Principal component analysis for the EXAFS spectra of Pt(II) adsorbed to goethite and hydrothermally-annealed goethite.

Component	Variance	Cumulative Variance	IND[a]
1	0.982428	0.982428	0.00665
2	0.004378	0.986805	0.00853
3	0.003995	0.990800	0.01122
4	0.003028	0.993828	0.01798
5	0.002367	0.996195	0.03595
6	0.002107	0.998302	0.12257
7	0.001698	1.000000	-

[a]Indicator value.

Table S6. Principal component analysis for the EXAFS spectra of Pt(II) adsorbed to hematite.

Component	Variance	Cumulative Variance	IND[a]
1	0.946589	0.946589	0.05616
2	0.028282	0.974871	0.05076
3	0.009868	0.984739	0.10183
4	0.008494	0.993233	0.34255
5	0.006767	1.000000	-

[a]Indicator value.

Table S7. Platinum L_3-edge EXAFS fitting parameters for hematite samples.

Sample[a]	[Cl] (mM)	Adsorbed Pt (μmol/m^2)[b]	Path	CN[c]	R (Å)[d]	σ^2 (Å^2)[e]	ΔE_0 (eV)[f]	*R* factor[g]	χ_v^2[g]
H0.5lPt	0.51	1.1	Pt-O	2.1(1)[h]	2.00(1)	0.0015(6)	11(1)	0.014	6.37
			Pt-Cl	1.9	2.285(7)	0.0015			
			Pt-Fe1[i]	0.2(1)	3.04(4)	0.002			
			Pt-Fe2	1.1(4)	3.69(2)	0.002			
			Pt-Fe3	1.0(4)	3.86(3)	0.002			
H0.5hPt	0.57	1.8	Pt-O	2.50(8)	2.010(7)	0.0010(4)	11.6(9)	0.011	6.58
			Pt-Cl	1.50	2.291(6)	0.0010			
			Pt-Fe1	0.2(1)	3.07(3)	0.002			
			Pt-Fe2	1.0(3)	3.71(2)	0.002			
			Pt-Fe3	0.9(4)	3.89(2)	0.002			
H2hPt	1.97	2.2	Pt-O	2.52(9)	2.020(8)	0.0010(5)	12(1)	0.013	10.17
			Pt-Cl	1.48	2.296(8)	0.0010			
			Pt-Fe1	0.3(1)	3.07(2)	0.002			
			Pt-Fe2	1.1(4)	3.72(2)	0.002			
			Pt-Fe3	0.9(4)	3.90(3)	0.002			
H10lPt	9.50	0.86	Pt-O	1.7(1)	2.02(1)	0.0019(5)	11.6(9)	0.007	1.72
			Pt-Cl	2.3	2.293(6)	0.0019			
			Pt-Fe1	0.3(1)	3.03(2)	0.002			
			Pt-Fe2	1.0(3)	3.70(2)	0.002			
			Pt-Fe3	0.8(3)	3.88(2)	0.002			
H10hPt	9.34	1.6	Pt-O	2.37(9)	2.014(8)	0.0008(5)	12(1)	0.013	5.29
			Pt-Cl	1.63	2.297(7)	0.0008			
			Pt-Fe1	0.3(1)	3.04(3)	0.002			
			Pt-Fe2	1.4(4)	3.73(2)	0.002			
			Pt-Fe3	1.0(4)	3.91(2)	0.002			

[a]See **Table S4** for more detailed sample information.
[b]Estimated by using the measured final aqueous Pt and calculating adsorbed Pt using the Langmuir isotherm values for the approximate chloride concentration (see **Table S2**).
[c]Coordination number.
[d]Interatomic distance.
[e]Debye-Waller factor.
[f]Energy shift parameter.
[g]Goodness-of-fit parameters (79).
[h]The estimated standard deviations as errors in the last digit are given in parentheses. Parameters that were constrained during fitting are listed with no uncertainties.
[i]Based on distances fit, Fe1 are edge-sharing neighbors, while Fe2 and Fe3 are corner-sharing neighbors.

Table S8. Platinum L_3-edge EXAFS fitting parameters for goethite and annealed goethite samples.

Sample[a]	[Cl] (mM)	Adsorbed Pt (μmol/m^2)[b]	Path	CN[c]	R (Å)[d]	σ^2 (Å^2)[e]	ΔE_0 (eV)[f]	*R* factor[g]	χ_v^2[g]
G0.5lPt	0.51	1.1	Pt-O	2.2(1)[h]	2.01(1)	0.0017(6)	11(1)	0.010	6.14
			Pt-Cl	1.8	2.273(8)	0.0017			
			Pt-Fe1[i]	0.1(1)	3.09(5)	0.002			
			Pt-Fe2	1.4(3)	3.68(1)	0.002			
			Pt-Fe3	1.1(4)	3.88(2)	0.002			
G0.5hPt	0.54	1.7	Pt-O	2.2(1)	2.009(8)	0.0015(5)	10.7(9)	0.007	7.86
			Pt-Cl	1.8	2.271(6)	0.0015			
			Pt-Fe1	0.17(9)	3.05(3)	0.002			
			Pt-Fe2	1.6(3)	3.68(1)	0.002			
			Pt-Fe3	1.3(3)	3.87(1)	0.002			
G2lPt	1.93	1.3	Pt-O	2.2(1)	2.01(1)	0.0015(5)	11(1)	0.010	17.42
			Pt-Cl	1.8	2.273(7)	0.0015			
			Pt-Fe1	0.1(1)	3.12(5)	0.002			
			Pt-Fe2	1.4(3)	3.68(1)	0.002			
			Pt-Fe3	1.0(4)	3.88(2)	0.002			
G2hPt	1.98	1.8	Pt-O	2.4(1)	2.02(1)	0.0012(6)	12(1)	0.011	17.80
			Pt-Cl	1.6	2.279(8)	0.0012			
			Pt-Fe1	0.1(1)	3.09(6)	0.002			
			Pt-Fe2	1.5(3)	3.68(1)	0.002			
			Pt-Fe3	1.2(4)	3.87(2)	0.002			
G10lPt	10.0	1.1	Pt-O	2.2(1)	2.02(1)	0.0014(5)	11(1)	0.008	7.81
			Pt-Cl	1.8	2.275(7)	0.0014			
			Pt-Fe1	0.1(1)	3.07(4)	0.002			
			Pt-Fe2	1.5(3)	3.68(1)	0.002			
			Pt-Fe3	1.1(3)	3.88(2)	0.002			
G10hPt	10.0	1.5	Pt-O	2.3(1)	2.013(9)	0.0013(5)	11(1)	0.009	10.61
			Pt-Cl	1.7	2.273(7)	0.0013			
			Pt-Fe1	0.2(1)	3.07(3)	0.002			
			Pt-Fe2	1.5(3)	3.68(1)	0.002			
			Pt-Fe3	1.1(4)	3.88(2)	0.002			
hG2Pt	2.14	1.6	Pt-O	2.2(1)	2.003(9)	0.0015(5)	10(1)	0.008	9.38
			Pt-Cl	1.8	2.270(7)	0.0015			
			Pt-Fe1	0.2(1)	3.06(3)	0.002			
			Pt-Fe2	1.4(3)	3.68(1)	0.002			
			Pt-Fe3	0.9(3)	3.88(2)	0.002			

[a]See **Table S4** for more detailed sample information.
[b]Estimated by using the measured final aqueous Pt and calculating adsorbed Pt using the Langmuir isotherm values for the non-annealed goethite isotherms at the approximate chloride concentration (see **Table S2**).
[c]Coordination number.
[d]Interatomic distance.
[e]Debye-Waller factor.
[f]Energy shift parameter.
[g]Goodness-of-fit parameters (79).
[h]The estimated standard deviations as errors in the last digit are given in parentheses. Parameters that were constrained during fitting are listed with no uncertainties.
[i]Based on distances fit, Fe1 are edge-sharing neighbors, while Fe2 and Fe3 are corner-sharing neighbors.

Table S9. Principal component analysis results for the EXAFS spectra of Pd(II) adsorbed to goethite and hydrothermally-annealed goethite.

Component	Variance	Cumulative Variance	IND[a]
1	0.946048	0.946048	0.01266
2	0.039880	0.985928	0.00242
3	0.003417	0.989346	0.00258
4	0.002649	0.991995	0.00307
5	0.002525	0.994520	0.00359
6	0.001951	0.996471	0.00429
7	0.001356	0.997826	0.00623
8	0.000999	0.998825	0.01042
9	0.000618	0.999443	0.03889
10	0.000557	1.000000	-

[a]Indicator value.

Table S10. Palladium K-edge EXAFS fitting parameters for goethite and annealed goethite samples.

Sample[a]	[Cl] (mM)	Adsorbed Pd (μmol/m²)[b]	Path	CN[c]	R (Å)[d]	σ^2 (Å²)[e]	ΔE_0 (eV)[f]	*R* factor[g]	χ_v^2[g]
G0.5lPd	0.49	0.80	Pd-O	2.31(8)[h]	2.013(7)	0.0019(4)	6(1)	0.010	34.90
			Pd-Cl	1.69	2.284(5)	0.0019			
			Pd-Fe4[i]	0.5(1)	2.94(1)	0.002			
			Pd-Fe5	0.5(1)	3.17(1)	0.002			
			Pd-Fe6	0.5(1)	3.59(2)	0.002			
G0.5hPd	0.48	1.0	Pd-O	2.57(6)	2.005(6)	0.0014(3)	5(1)	0.012	10.54
			Pd-Cl	1.43	2.281(5)	0.0014			
			Pd-Fe4	0.6(1)	2.94(1)	0.002			
			Pd-Fe5	0.9(2)	3.17(1)	0.002			
			Pd-Fe6	0.4(2)	3.57(2)	0.002			
G2lPd	1.75	1.2	Pd-O	2.11(9)	2.010(7)	0.0012(4)	6(1)	0.012	11.60
			Pd-Cl	1.89	2.280(5)	0.0012			
			Pd-Fe4	0.5(1)	2.94(2)	0.002			
			Pd-Fe5	0.6(2)	3.16(2)	0.002			
			Pd-Fe6	0.7(2)	3.60(1)	0.002			
G2hPd	1.88	1.9	Pd-O	2.82(6)	2.004(5)	0.0017(3)	5(1)	0.009	26.78
			Pd-Cl	1.18	2.282(6)	0.0017			
			Pd-Fe4	0.8(1)	2.928(7)	0.002			
			Pd-Fe5	1.1(1)	3.167(7)	0.002			
			Pd-Fe6	0.5(1)	3.57(2)	0.002			
G10lPd	11.0	0.62	Pd-O	1.9(1)	2.021(9)	0.0017(4)	7(1)	0.015	81.30
			Pd-Cl	2.1	2.284(5)	0.0017			
			Pd-Fe6	0.6(2)	3.62(2)	0.002			
G10hPd	10.2	1.5	Pd-O	2.33(8)	2.009(7)	0.0011(4)	6(1)	0.015	33.55
			Pd-Cl	1.67	2.284(5)	0.0011			
			Pd-Fe4	0.4(1)	2.93(2)	0.002			
			Pd-Fe5	0.6(2)	3.17(2)	0.002			
			Pd-Fe6	0.7(2)	3.61(2)	0.002			
hG0.5Pd	0.50	1.3	Pd-O	2.73(6)	2.007(6)	0.0012(3)	6(1)	0.014	23.03
			Pd-Cl	1.27	2.286(6)	0.0012			
			Pd-Fe4	0.6(1)	2.94(1)	0.002			
			Pd-Fe5	0.9(2)	3.18(1)	0.002			
			Pd-Fe6	0.4(2)	3.555(3)	0.002			
hG2lPd	1.82	1.4	Pd-O	2.58(5)	2.008(5)	0.0012(3)	6(1)	0.007	3.00
			Pd-Cl	1.42	2.281(4)	0.0012			
			Pd-Fe4	0.5(1)	2.93(1)	0.002			
			Pd-Fe5	0.8(1)	3.168(8)	0.002			
			Pd-Fe6	0.6(1)	3.57(1)	0.002			
hG2hPd	1.98	2.0	Pd-O	2.90(6)	2.006(5)	0.0014(3)	6(1)	0.013	61.98
			Pd-Cl	1.10	2.288(7)	0.0014			
			Pd-Fe4	0.8(1)	2.928(9)	0.002			
			Pd-Fe5	1.1(2)	3.171(8)	0.002			
			Pd-Fe6	0.4(2)	3.56(2)	0.002			
hG10Pd	10.3	1.5	Pd-O	2.33(8)	2.010(7)	0.0011(4)	6(1)	0.015	32.68
			Pd-Cl	1.67	2.286(5)	0.0011			
			Pd-Fe4	0.4(2)	2.93(2)	0.002			
			Pd-Fe5	0.5(2)	3.16(2)	0.002			
			Pd-Fe6	0.6(2)	3.60(2)	0.002			

[a]See **Table S4** for more details about samples.
[b]Estimated by using the measured final aqueous Pd and calculating adsorbed Pd using the Langmuir isotherm values for the non-annealed goethite isotherms at the approximate chloride concentration (see **Table S2**).
[c]Coordination number.
[d]Interatomic distance.

[e]Debye-Waller factor.
[f]Energy shift parameter.
[g]Goodness-of-fit parameters (79).
[h]The estimated standard deviations as errors in the last digit are given in parentheses. Parameters that were constrained during fitting are listed with no uncertainties.
[i] Based on distances fit, Fe4 and Fe5 are edge-sharing neighbors, while Fe 6 is a corner-sharing neighbor.

Table S11. Palladium K-edge EXAFS fitting parameters for hematite samples.

Sample[a]	[Cl] (mM)	Adsorbed Pd (μmol/m²)[b]	Path	CN[c]	R (Å)[d]	σ^2 (Å²)[e]	ΔE_0 (eV)[f]	*R* factor[g]	χ_v^2[g]
H0.5hPd	0.43	0.50	Pd-O	2.1(1)[h]	2.012(9)	0.0026(4)	5(1)	0.013	23.86
			Pd-Cl	1.9	2.283(6)	0.0026			
			Pd-Fe4[i]	0.3(1)	2.911(3)	0.002			
			Pd-Fe5	0.3(2)	3.07(3)	0.002			
			Pd-Fe6	0.3(2)	3.68(3)	0.002			
H5hPd	4.7	0.88	Pd-O	1.7(1)	2.01(1)	0.0020(4)	6(1)	0.015	46.62
			Pd-Cl	2.3	2.297(5)	0.0020			
			Pd-Fe4	0.2(2)	2.91(5)	0.002			
			Pd-Fe5	0.2(2)	3.08(4)	0.002			
			Pd-Fe6	0.3(2)	3.65(4)	0.002			
H10lPd	9.7	0.29	Pd-O	1.5(1)	2.014(9)	0.0021(4)	6.3(8)	0.009	20.53
			Pd-Cl	2.5	2.298(4)	0.0021			
			Pd-Fe4	0.2(1)	2.91(4)	0.002			
			Pd-Fe5	0.2(2)	3.10(4)	0.002			
			Pd-Fe6	0.2(2)	3.66(4)	0.002			
H10hPd	10	1.1	Pd-O	1.4(2)	2.02(1)	0.0025(4)	7(1)	0.011	62.93
			Pd-Cl	2.6	2.303(5)	0.0025			
			Pd-Fe4	0.4(2)	2.93(2)	0.002			
			Pd-Fe5	0.2(2)	3.10(4)	0.002			
			Pd-Fe6	0.3(2)	3.61(3)	0.002			

[a]See Wright et al. (2) for more details about samples.
[b]Estimated by using the measured final aqueous Pd and calculating adsorbed Pd using the Langmuir isotherm values for the hematite isotherms at the approximate chloride concentration (see Wright et al. (2)).
[c]Coordination number.
[d]Interatomic distance.
[e]Debye-Waller factor.
[f]Energy shift parameter.
[g]Goodness-of-fit parameters (79).
[h]The estimated standard deviations as errors in the last digit are given in parentheses. Parameters that were constrained during fitting are listed with no uncertainties.
[i] Based on distances fit, Fe4 and Fe5 are edge-sharing neighbors, while Fe 6 is a corner-sharing neighbor.

SI References

1. U. Schwertmann, R. M. Cornell, *Iron Oxides in the Laboratory: Preparation and Characterization* (Wiley-VCH, Weinheim, ed. 2, 2000).
2. E. G. Wright, X. He, E. D. Flynn, D. E. Giammar, J. G. Catalano, Competitive and cooperative effects of chloride on palladium(II) adsorption to iron (oxyhydr)oxides: Implications for mobility during weathering. *Geochim. Cosmochim. Acta* **391**, 203-217 (2025).
3. R. G. McGregor, D. W. Blowes, J. L. Jambor, W. D. Robertson, The solid-phase controls on the mobility of heavy metals at the Copper Cliff tailings area, Sudbury, Ontario, Canada. *J. Contam. Hydrol.* **33**, 247-271 (1998).
4. A. Alexander, J. Ndambuki, R. Salim, A. Manda, Assessment of Spatial Variation of Groundwater Quality in a Mining Basin. *Sustainability* **9**, 823 (2017).
5. D. P. Ahokpossi, A. Atangana, P. D. Vermeulen, Hydro-geochemical characterizations of a platinum group element groundwater system in Africa. *J. Afr. Earth Sci.* **138**, 348-366 (2018).
6. M. Molekoa, R. Avtar, P. Kumar, H. Minh, T. Kurniawan, Hydrogeochemical Assessment of Groundwater Quality of Mokopane Area, Limpopo, South Africa Using Statistical Approach. *Water* **11**, 1891 (2019).
7. W. B. Coker *et al.*, The behaviour of platinum group elements in the surficial environment at Ferguson Lake, N.W.T., Rottenstone Lake, Sask. and Sudbury, Ont., Canada. *J. Geochem. Explor.* **40**, 165-192 (1991).
8. A. S. Mashio *et al.*, Dissolved platinum in rainwater, river water and seawater around Tokyo Bay and Otsuchi Bay in Japan. *Estuar. Coast. Shelf Sci.* **180**, 160-167 (2016).
9. A. Cobelo-García, M. E. Mulyani, J. Schäfer, Ultra-trace interference-free analysis of palladium in natural waters by ICP-MS after on-line matrix separation and pre-concentration. *Talanta* **232**, 122289 (2021).
10. L. G. M. Baas Becking, I. R. Kaplan, D. Moore, Limits of the Natural Environment in Terms of pH and Oxidation-Reduction Potentials. *J. Geol.* **68**, 243-284 (1960).
11. R. C. Thompson, K. A. Rodgers, Laterization of the Ultramafic-Gabbro Association at North Cape, Northernmost New Zealand. *J. R. Soc. N. Z.* **7**, 347-377 (1977).
12. D. Fandeur *et al.*, XANES Evidence for Oxidation of Cr(III) to Cr(VI) by Mn-Oxides in a Lateritic Regolith Developed on Serpentinized Ultramafic Rocks of New Caledonia. *Environ. Sci. Technol.* **43**, 7384-7390 (2009).
13. A. Ito *et al.*, Geochemical constraints on the mobilization of Ni and critical metals in laterite deposits, Sulawesi, Indonesia: A mass-balance approach. *Resour. Geol.* **71**, 255-282 (2021).
14. C. Colombo, C. J. Oates, A. J. Monhemius, J. A. Plant, Complexation of platinum, palladium and rhodium with inorganic ligands in the environment. *Geochem.: Explor. Environ. Anal.* **8**, 91-101 (2008).
15. S. A. Wood, The role of humic substances in the transport and fixation of metals of economic interest (Au, Pt, Pd, U, V). *Ore Geol. Rev.* **11**, 1-31 (1996).
16. S. A. Wood, C. D. Tait, D. Vlassopoulos, D. R. Janecky, Solubility and spectroscopic studies of the interaction of palladium with simple carboxylic acids and fulvic acid at low temperature. *Geochim. Cosmochim. Acta* **58**, 625-637 (1994).
17. S. A. Wood, J. V. Middlesworth, The influence of acetate and oxalate as simple organic ligands on the behavior of palladium in surface environments. *Can. Mineral.* **42**, 411-421 (2004).
18. A. N. Awolayo, B. M. Tutolo, PyGeochemCalc: A Python package for geochemical thermodynamic calculations from ambient to deep Earth conditions. *Chem. Geol.* **606**, 120984 (2022).
19. J. M. Delany, S. R. Lundeen (1990) The LLNL thermochemical database. Lawrence Livermore National Laboratory Report UCRL-21658, 150 p.
20. J. P. Gustafsson (2022) Visual MINTEQ Version 4.0. http://vminteq.com.
21. D. L. Parkhurst, C. A. J. Appelo, "Description of input and examples for PHREEQC version 3—A computer program for speciation, batch-reaction, one-dimensional transport, and inverse geochemical calculations" in U.S. Geological Survey Techniques and Methods, book 6. (2013), chap. A43. https://doi.org/10.3133/tm6A43.
22. E. Colàs *et al.*, The ThermoChimie database: Comparison with other databases and example calculations for radionuclides and chemo-toxic elements. *Appl. Geochem.* **179** (2025).

23. M. Azaroual, B. Romand, P. Freyssinet, J.-R. Disnar, Solubility of platinum in aqueous solutions at 25°C and pHs 4 to 10 under oxidizing conditions. *Geochim. Cosmochim. Acta* **65**, 4453-4466 (2001).
24. M. Azaroual, B. Romand, P. Freyssinet, J.-R. Disnar, Response to the comment by R. H. Byrne on "Solubility of platinum in aqueous solutions at 25°C and pHs 4 to 10 under oxidizing conditions" (2001) Geochim. Cosmochim. Acta 65, 4453–4466. *Geochim. Cosmochim. Acta* **67**, 2511-2513 (2003).
25. R. H. Byrne, Comment on "Solubility of platinum in aqueous solutions at 25°C and pHs 4 to 10 under oxidizing conditions" by Mohamed Azaroual, Bruno Romand, Philippe Freyssinet, and Jean-Robert Disnar. *Geochim. Cosmochim. Acta* **67**, 2511-2513 (2003).
26. B. M. Mountain, S. A. Wood, Chemical Controls on the Solubility, Transport, and Deposition of Platinum and Palladium in Hydrothermal Solutions: A Thermodynamic Approach. *Econ. Geol.* **83**, 492-510 (1988).
27. S. A. Wood, B. M. Mountain, B. J. Fenlon, Thermodynamic Constraints on the Solubility of Platinum and Palladium in Hydrothermal Solutions: Reassessment of Hydroxide, Bisulfide, and Ammonia Complexing. *Econ. Geol.* **84**, 2020-2028 (1989).
28. D. C. Sassani, E. L. Shock, Solubility and transport of platinum-group elements in supercritical fluids: Summary and estimates of thermodynamic properties for ruthenium, rhodium, palladium, and platinum solids, aqueous ions, and complexes to 1000°C and 5 kbar. *Geochim. Cosmochim. Acta* **62**, 2643-2671 (1998).
29. D. Rai, M. Yui, A. Kitamura, Thermodynamic Model for Amorphous $Pd(OH)_2$ Solubility in the Aqueous Na^+–K^+–H^+–OH^-–Cl^-–ClO_4^-–H_2O System at 25°C: A Critical Review. *J. Solution Chem.* **41**, 1965-1985 (2012).
30. D. C. Sassani, E. L. Shock, Speciation and solubility of palladium in aqueous magmatic-hydrothermal solutions. *Geology* **18**, 925-928 (1990).
31. B. R. Tagirov *et al.*, The speciation and transport of palladium in hydrothermal fluids: Experimental modeling and thermodynamic constraints. *Geochim. Cosmochim. Acta* **117**, 348-373 (2013).
32. M. Newville, IFEFFIT: interactive XAFS analysis and FEFF fitting. *J. Synchrotron Rad.* **8**, 322-324 (2001).
33. S. M. Webb, SIXpack: a graphical user interface for XAS analysis using IFEFFIT. *Phys. Scr.* **T115**, 1011-1014 (2005).
34. B. Ravel, M. Newville, ATHENA, ARTEMIS, HEPHAESTUS: data analysis for X-ray absorption spectroscopy using IFEFFIT. *J. Synchrotron Radiat.* **12**, 537-541 (2005).
35. A. L. Ankudinov, B. Ravel, J. J. Rehr, S. D. Conradson, Real-space multiple-scattering calculation and interpretation of x-ray-absorption near-edge structure. *Phys. Rev. B* **58**, 7565-7576 (1998).
36. M. Newville, Larch: An Analysis Package for XAFS and Related Spectroscopies. *J. Phys.: Conf. Ser.* **430**, 012007 (2013).
37. B. Dannecker, G. Thiele, Darstellung und Kristallstruktur von Palladiumoxidchlorid Pd_2OCl_2. *Z. Naturforsch* **41b**, 1363-1366 (1986).
38. J. Arpalahti, R. Sillanpää, M. Mikola, Facile Isolation and Crystal Structure Determination of *trans*-$[PtCl(OH)(NH_3)_2]\cdot H_2O$. *J. Chem. Soc., Dalton Trans.* **9**, 1499-1500 (1994).
39. R. Zeese, U. Schwertmann, G. F. Tietz, U. Jux, Mineralogy and stratigraphy of three deep lateritic profiles of the Jos plateau (Central Nigeria). *Catena* **21**, 195-214 (1994).
40. I. Giorgis *et al.*, The lateritic profile of Balkouin, Burkina Faso: Geochemistry, mineralogy and genesis. *J. Afr. Earth Sci.* **90**, 31-48 (2014).
41. P. H. C. d. Santos, M. L. d. Costa, A. S. Leite, The Piriá aluminous lateritic profile: mineralogy, geochemistry and parent rock. *Braz. J. Geol.* **46**, 617-636 (2016).
42. A. L. Giovannini *et al.*, Mineralogy and geochemistry of laterites from the Morro dos Seis Lagos Nb (Ti, REE) deposit (Amazonas, Brazil). *Ore Geol. Rev.* **88**, 461-480 (2017).
43. D. Domínguez-Carretero *et al.*, The Geology, Geochemistry, and Mineralogy of the Moa Bay Ni Laterite Mining District, Cuba. *Econ. Geol.* **119**, 1685-1706 (2024).
44. E. Tauler *et al.*, Geochemistry and Mineralogy of the Clay-Type Ni-Laterite Deposit of San Felipe (Camagüey, Cuba). *Minerals* **13** (2023).

45. E. Fritsch *et al.*, Transformation of haematite and Al-poor goethite to Al-rich goethite and associated yellowing in a ferralitic clay soil profile of the middle Amazon Basin (Manaus, Brazil). *Eur. J. Soil Sci.* **56**, 575-588 (2005).
46. E. Fritsch *et al.*, Deciphering the weathering processes using environmental mineralogy and geochemistry: Towards an integrated model of laterite and podzol genesis in the Upper Amazon Basin. *C. R. - Geosci.* **343**, 188-198 (2011).
47. M. Elias, M. J. Donaldson, N. Giorgetta, Geology, Mineralogy, and Chemistry of Lateritic Nickel-Cobalt Deposits near Kalgoorlie, Western Australia. *Econ. Geol.* **76**, 1775-1783 (1981).
48. G. Dublet *et al.*, Goethite aging explains Ni depletion in upper units of ultramafic lateritic ores from New Caledonia. *Geochim. Cosmochim. Acta* **160**, 1-15 (2015).
49. Y. Choi, I. Lee, I. Moon, Geochemical and Mineralogical Characteristics of Garnierite From the Morowali Ni-Laterite Deposit in Sulawesi, Indonesia. *Front. Earth Sci.* **9** (2021).
50. D. J. Gray, K. H. Schorin, C. R. M. Butt, Mineral associations of platinum and palladium in lateritic regolith, Ora Banda Sill, Western Australia. *J. Geochem. Explor.* **57**, 245-255 (1996).
51. M. Locmelis, F. Melcher, T. Oberthür, Platinum-group element distribution in the oxidized Main Sulfide Zone, Great Dyke, Zimbabwe. *Miner. Deposita* **45**, 93-109 (2010).
52. Y. Takahashi, Y. Minai, S. Ambe, Y. Makide, F. Ambe, Comparison of adsorption behavior of multiple inorganic ions on kaolinite and silica in the presence of humic acid using the multitracer technique. *Geochim. Cosmochim. Acta* **63**, 815-836 (1999).
53. T. Aiglsperger *et al.*, Platinum group minerals (PGM) in the Falcondo Ni-laterite deposit, Loma Caribe peridotite (Dominican Republic). *Miner. Deposita* **50**, 105-123 (2015).
54. K. A. Aquino, C. A. Arcilla, C. Schardt, C. A. J. Tupaz, Mineralogical and Geochemical Characterization of the Sta. Cruz Nickel Laterite Deposit, Zambales, Philippines. *Minerals* **12** (2022).
55. U. Schwertmann, N. Kämpf, Properties of Goethite and Hematite in Kaolinitic Soils of Southern and Central Brazil. *Soil Sci.* **139**, 344-350 (1985).
56. R. R. Anand, R. J. Gilkes, Variations in the Properties of Iron Oxides within Individual Specimens of Lateritic Duricrust. *Aust. J. Soil Res.* **25**, 287-302 (1987).
57. L. J. Kirwan, F. A. Deeney, G. M. Croke, K. Hodnett, Characterisation of various Jamaican bauxite ores by quantitative Rietveld X-ray powder diffraction and ^{57}Fe Mössbauer spectroscopy. *Int. J. Miner. Process.* **91**, 14-18 (2009).
58. U. Schwertmann, M. Latham, Properties of Iron Oxides in Some New Caledonian Oxisols. *Geoderma* **39**, 105-123 (1986).
59. M. L. M. d. Carvalho-e-Silva *et al.*, Characterization of Ni-Containing Goethites by Mössbauer Spectroscopy and Other Techniques. *Hyperfine Interact.* **142**, 559-576 (2002).
60. G. Matheis, M. J. Pearson, Mineralogy and Geochemical Dispersion in Lateritic Soil Profiles of Northern Nigeria. *Chem. Geol.* **35**, 129-145 (1982).
61. T. G. St. Pierre, J. Webb, C. R. M. Butt, Lateritic Mineralization near Kalgoorlie, Western Australia: Dating by Mössbauer Spectroscopy? *Hyperfine Interact.* **57**, 2279-2284 (1990).
62. S. M. B. d. Oliveira, C. S. M. Partiti, J. Enzweiler, Ochreous laterite: a nickel ore from Punta Gorda, Cuba. *J. South Am. Earth Sci.* **14**, 307-317 (2001).
63. C. Van Cromphaut *et al.*, Characterization by Mössbauer spectroscopy of Fe phases in highly weathered serpentinitic soil from southern Cameroon. *Clay Miner.* **43**, 117-128 (2008).
64. D. Traoré *et al.*, Platinum and palladium mobility in supergene environment: The residual origin of the Pirogues River mineralization, New Caledonia. *J. Geochem. Explor.* **88**, 350-354 (2006).
65. S. Suárez, H. M. Prichard, F. Velasco, P. C. Fisher, I. McDonald, Alteration of platinum-group minerals and dispersion of platinum-group elements during progressive weathering of the Aguablanca Ni–Cu deposit, SW Spain. *Miner. Deposita* **45**, 331-350 (2010).
66. D. G. Eliopoulos, M. Economou-Eliopoulos, Geochemical and mineralogical characteristics of Fe-Ni- and bauxitic-laterite deposits of Greece. *Ore Geol. Rev.* **16**, 41-58 (2000).
67. P.-D. Ndjigui, P. Bilong, Platinum-group elements in the serpentinite lateritic mantles of the Kongo–Nkamouna ultramafic massif (Lomié region, South-East Cameroon). *J. Geochem. Explor.* **107**, 63-76 (2010).
68. M. Villalobos, M. A. Trotz, J. O. Leckie, Variability in goethite surface site density: evidence from proton and carbonate sorption. *J. Colloid Interface Sci.* **268**, 273-287 (2003).

69. M. Y. Maeno *et al.*, Sorption behavior of the Pt(II) complex anion on manganese dioxide (δ-MnO_2): a model reaction to elucidate the mechanism by which Pt is concentrated into a marine ferromanganese crust. *Miner. Deposita* **51**, 211-218 (2015).
70. A. Koschinsky *et al.*, Platinum enrichment and phase associations in marine ferromanganese crusts and nodules based on a multi-method approach. *Chem. Geol.* **539** (2020).
71. E. F. Bazarkina, G. S. Pokrovski, J.-L. Hazemann, Structure, stability and geochemical role of palladium chloride complexes in hydrothermal fluids. *Geochim. Cosmochim. Acta* **146**, 107-131 (2014).
72. K. Tanaka, M. Tanaka, N. Watanabe, K. Tokunaga, Y. Takahashi, Ligand exchange adsorption and coordination structure of Pd on δ-MnO_2 in NaCl solution. *Chem. Geol.* **460**, 130-137 (2017).
73. L. D. Troyer *et al.*, Effect of phosphate on U(VI) sorption to montmorillonite: Ternary complexation and precipitation barriers. *Geochim. Cosmochim. Acta* **175**, 86-99 (2016).
74. S. Bocquet, A. J. Hill, Correlation of Néel Temperature and Vacancy Defects in Fine-Particle Goethites. *Phys. Chem. Minerals* **22**, 524-528 (1995).
75. N. Taitel-Goldman, C. Bender Koch, A. Singer, Si-Associated Goethite in Hydrothermal Sediments of the Atlantis II and Thetis Deeps, Red Sea. *Clays Clay Miner.* **52**, 115-129 (2004).
76. S. D. Taylor *et al.*, Visualizing the iron atom exchange front in the Fe(II)-catalyzed recrystallization of goethite by atom probe tomography. *Proc. Natl. Acad. Sci. U.S.A.* **116**, 2866-2874 (2019).
77. L. Notini *et al.*, The Role of Defects in Fe(II)-Goethite Electron Transfer. *Environ. Sci. Technol.* **52**, 2751-2759 (2018).
78. M. Ishii, XAFS spectrum of Palladium(II) oxide hydrous. Materials Data Repository (2021), https://doi.org/10.48505/nims.2256.
79. S. D. Kelly, D. Hesterberg, B. Ravel, "Analysis of Soils and Minerals Using X-ray Absorption Spectroscopy" in Methods of Soil Analysis Part 5—Mineralogical Methods, A. L. Ulery, L. R. Drees, Eds. (Soil Science of America, Inc., Madison, 2008), vol. 5, chap. 14, pp. 387-464.
